# Vertically stratified carbon fixation and coupling processes in deep-sea sediment

**DOI:** 10.1093/ismeco/ycaf242

**Published:** 2025-12-18

**Authors:** Hai Shi, Xiaotong Zhang, Liyan Liu, Fabiano Thompson, Xueqi Li, Haowei Sun, Huichao Mi, Xiao-Hua Zhang, Yunhui Zhang

**Affiliations:** Frontiers Science Center for Deep Ocean Multispheres and Earth System, and College of Marine Life Sciences, Ocean University of China, No. 5 Yushan Road, Shinan District, Qingdao 266000, China; Frontiers Science Center for Deep Ocean Multispheres and Earth System, and College of Marine Life Sciences, Ocean University of China, No. 5 Yushan Road, Shinan District, Qingdao 266000, China; Frontiers Science Center for Deep Ocean Multispheres and Earth System, and College of Marine Life Sciences, Ocean University of China, No. 5 Yushan Road, Shinan District, Qingdao 266000, China; Institute of Biology and Coppe, Federal University of Rio de Janeiro (UFRJ), Avenida Pedro Calmon, nº 550, Cidade Universitária, Rio 21941-599, Brazil; Frontiers Science Center for Deep Ocean Multispheres and Earth System, and College of Marine Life Sciences, Ocean University of China, No. 5 Yushan Road, Shinan District, Qingdao 266000, China; Frontiers Science Center for Deep Ocean Multispheres and Earth System, and College of Marine Life Sciences, Ocean University of China, No. 5 Yushan Road, Shinan District, Qingdao 266000, China; Frontiers Science Center for Deep Ocean Multispheres and Earth System, and College of Marine Life Sciences, Ocean University of China, No. 5 Yushan Road, Shinan District, Qingdao 266000, China; Frontiers Science Center for Deep Ocean Multispheres and Earth System, and College of Marine Life Sciences, Ocean University of China, No. 5 Yushan Road, Shinan District, Qingdao 266000, China; Laboratory for Marine Ecology and Environmental Science, Qingdao Marine Science and Technology Center, No. 168 Wenhai Middle Road, Jimo District, Qingdao 266000, China; Key Laboratory of Evolution & Marine Biodiversity (Ministry of Education) and Institute of Evolution & Marine Biodiversity, Ocean University of China, No. 5 Yushan Road, Shinan District, Qingdao 266000, China; Key Laboratory of Evolution & Marine Biodiversity (Ministry of Education) and Institute of Evolution & Marine Biodiversity, Ocean University of China, No. 5 Yushan Road, Shinan District, Qingdao 266000, China

**Keywords:** deep-sea sediment, carbon fixation, vertical stratification, redox processes

## Abstract

Deep-sea sediments represent a vast yet underexplored reservoir of microbial carbon fixation, playing a critical role in global carbon cycling. However, the vertical distribution of carbon-fixing microorganisms, metabolic pathways, and the underlying energy sources and environmental drivers remain poorly understood. In this study, we investigated microbial carbon fixation and associated energy metabolism in South China Sea (SCS) sediment across 0–690 cm depth. Our findings revealed that dissolved inorganic carbon (DIC) and ammonium (NH₄^+^) concentrations were key environmental drivers of carbon fixation and linked redox processes. Carbon fixation gene diversity increased with sediment depth, while the network complexity of functional genes and taxa involved in these processes declined. A distinct vertical succession of dominant microbial carbon-fixation pathways and their associated energy metabolisms was observed along the sediment depth: the Calvin-Benson-Bassham (CBB) and reductive glycine (rGLY) pathways dominated surface sediments, driven by nitrite oxidation, whereas the Wood-Ljungdahl (WL) pathway prevailed in deeper anoxic layers, supported by hydrogen and carbon monoxide oxidation. Taxonomically, Gammaproteobacteria and Methylomirabilia were abundant carbon-fixing groups in surface sediments, while Desulfobacterota, Chloroflexota, and Aerophobota became predominant at depth. Most carbon-fixing metagenome-assembled genomes (MAGs) exhibited mixotrophic lifestyles, and representative carbon fixation MAGs from Methylomirabilota, Dehalococcoidia (Chloroflexota) and Aerophobetes exhibited different metabolic features compared to their counterparts from other environments. These findings underscore the carbon fixation potential of deep-sea subsurface microbial communities and advance the understanding of carbon fluxes in deep biosphere.

## Introduction

Covering ~65% of Earth's surface, deep-sea sediments serve as a repository of organic and inorganic materials and play a key role in global biogeochemical cycles [1, 2]. In these benthic ecosystems, bacteria and archaea dominate the biomass and drive critical processes such as carbon sequestration, nutrient cycling, and energy transfer [3–7]. Globally, deep-sea sediments are vital hotspots for carbon remineralization and burial [8, 9]. The sequestration of microbially transformed organic matter is the primary mechanism of carbon preservation in sediments [9, 10], while carbon fixation also actively occurs alongside remineralization. Through chemolithoautotrophic processes, marine microbes fix up to 370 Tg C/year—a rate comparable to annual organic carbon burial—and accounting for 48% of chemolithoautotrophic carbon fixation in the ocean [11]. Almost half of this fixed carbon occurs in shallow, near-shore sediments, which have been the primary focus of most studies on marine sediment carbon fixation [12–15]. However, deep-sea sediments, such as those on continental slope, may also make an indispensable contribution to carbon fixation [11], where chemoautotrophic microbes thrive along such redox gradient systems [13]. It is estimated that inorganic carbon fixation accounted for 19% of the total heterotrophic biomass production in the surface deep-sea sediments, supporting the functioning of the benthic food webs in the deep-sea [16]. Despite their importance, a thorough understanding of how microbial activities drive carbon fixation along depth-dependent redox gradients in deep-sea sediments has yet to be achieved.

Currently, eight pathways of autotrophic carbon fixation have been identified [17], including the Calvin-Benson-Bassham (CBB) cycle, the 3-hydroxypropionate/4-hydroxybutyrate (3-HP/4-HB) cycle, reductive tricarboxylic acid (rTCA) cycle, reductive acetyl-CoA (Wood-Ljungdahl, WL) pathway, 3-hydroxypropionate (3-HP) cycle, dicarboxylate/4-hydroxybutyrate (DC/4-HB) cycle, the reductive glycine (rGly) cycle and the reverse oxidative TCA (roTCA) cycle [13, 14]. Among them, the CBB cycle is the most widespread and extensively studied pathway in marine coastal sediments and other habitats [12, 14]. Some of these carbon fixation pathways, such as the rTCA cycle, are found across diverse bacterial and archaeal phyla [18], while others are thought to occur only in particular taxa (e.g. the 3-HP/4-HB cycle predominantly restricted to archaea [19]). However, the relative abundance of these distinct pathways, the specific microbial communities encoding them, and their vertical stratification patterns within deep-sea sediments remain to be elucidated.

The inorganic carbon fixation is closely associated with the cycles of nitrogen, sulfur, hydrogen and metals [20]. These reductive and oxidative processes are highly active in deep-sea sediments along the redox gradients, supplying abundant substrates for chemoautotrophy. Nitrification is one of the main processes responsible for dark carbon fixation in the ocean [21], with nitrifying bacteria and archaea widely distributed across diverse taxa. Among these processes, carbon fixation driven by aerobic ammonia oxidation in archaea is considered significantly contribute to sustain the prokaryotic heterotrophic carbon requirements in deep sea [22, 23]. Inorganic reduced sulfur compounds, such as sulfide and thiosulfate, also serve as important drivers for chemoautotrophic carbon fixation [24], which may exist in large amounts as the products of sulfate reduction in deep-sea sediments [25]. In coastal sediments, sulfur-oxidizing Gammaproteobacteria contribute to 70%–86% of carbon fixation [12]. Additionally, other potential important energy sources for carbon fixation include oxidation of iron, H_2_, CO, and CH_4_ [26–29]. Therefore, disentangling the vertical succession of these processes in deep-sea sediments and their coupling with carbon fixation is fundamental for uncovering the microbial mechanisms that regulate carbon turnover in the deep biosphere.

In a previous study, we collected deep-sea (1250–3530 m) sediment cores of ~7 m and provided a highly resolved vertical profile of microbial abundance and distribution in the continental slope of South China Sea (SCS) [30]. Microbial communities in these samples exhibited clear vertical succession consistent with redox zonation, and increased dissolved inorganic carbon (DIC) along sediment depths was observed [30]. Therefore, such a representative deep-sea sediment environment could be ideal to study microbial dark carbon fixation and its coupling processes. In this study, we performed comprehensive metagenomic analyses on these sediment samples, aiming to provide novel insights into the vertical changes and coupling of carbon fixation pathways with other important processes, and to uncover the unique carbon fixation microbial taxa and their specific coupling mechanisms in deep-sea sediments.

## Materials and methods

### Sediment sample collection

The sediment samples were collected by a gravity corer during cruise HYSH201901 organized by Guangzhou Marine Geological Survey from February 26 to March 6, 2019 in our previous study [30]. Based on the location and microbial community composition, three representative sediment cores from 1250 to 3530 water depth were chosen for metagenomic sequencing (SCS003, SCS008, and SCS011, 18.44–19.26°N, 114.25–115.30°E, Fig. S1). Each core was subsampled from six depth intervals (0, 5, 50, 190, 390, and 690 cm), resulting in 18 samples in total. The sediments were transferred into sterile plastic tubes and stored at −80°C prior to DNA extraction. The collection of porewater samples, measurement of total OC (TOC), total nitrogen (TN), DIC, and nutrients and other solutes (NH_4_^+^, NO_3_^−^, NO_2_^−^, SO_4_^2−^, C/N) were performed as described by Zhang et al. [30].

### DNA extraction and metagenome sequencing

Genomic DNA was extracted and purified with the DNeasy® PowerClean® Pro Cleanup Kit (50) (QIAGEN, catalog number: 12997–50). DNA quality was assessed using a Nanodrop ND-FISHER 2000 spectrophotometer. Metagenome sequencing was performed at BGI Tech Solutions (Beijing Liuhe) Co., Ltd., and Guangdong Magigene Biotechnology (Guangzhou, China) Co., Ltd.. All libraries were sequenced on the MGI-SEQ-T7 platform (PE150), yielding ~50 Gb of high-quality data per sample (Table S1).

### Metagenome sequence assembly

Raw reads were filtered using MetaWRAP's read_qc module with default parameters, which perform quality trimming via Trim Galore (cutoff′-q 20′) and generate quality reports using FastQC, to obtain clean reads [31]. Clean reads were assembled into contigs with metaSPAdes (v3.15.4) [32] using default parameters, and genes were predicted via Prodigal (v2.6.3) [33]. Redundant sequences were removed using Cd-hit (−c 0.95 -n 10 -d 0 -M 0 -aS 0.9; v4.8.1) to generate a nonredundant gene set [34]. Gene relative abundances were calculated by mapping reads with BWA (v0.7.18-r1243-dirty) [35], quantifying with CoverM (v0.6.1; −min-read-aligned-percent-pair 80 --proper-pairs-only) [36], and processing with Samtools (v1.18) [37], then normalized to transcripts per million transcripts (TPM). Predicted proteins were annotated first with Metabolic (v4.0) [38]. For identification of metabolic marker genes not covered by the standard Metabolic workflow, protein sequences were searched against the KEGG HMM collection using HMMER (v3.1b2). Only hits meeting the per-profile cutoffs provided with the KEGG HMMs (KEGG-recommended default thresholds) were retained. Functional genes involved in carbon fixation and related pathways were extracted from the nonredundant set. For the GCV gene cluster in the rGly pathway and the kor and por gene clusters in the roTCA pathway, relative abundance was averaged across each gene cluster. Oxidative and reductive *dsrAB* genes were distinguished by comparison to a curated *dsrAB* database [39] and phylogenetic tree construction (Fig. S2). Representative metabolic genes are listed in Table S2, and their vertical distribution and ecological roles were inferred from relative abundance profiles.

Amino acid sequences of functional genes were extracted using SeqKit (v2.5.1) [40] and aligned to the nr database with DIAMOND (v0.9.19.120) [41]. Taxonomic annotation information was then retrieved using MEGAN (v6.25.9) [42] to classify key genes and to calculate their abundance at the genus level.

### Analysis of environmental factors driving carbon fixation

First, for each metabolic pathway, gene counts per sample were summed and converted to TPM to produce a pathway × sample relative-abundance matrix; pathway-associated sequences were annotated to genus and genus-level counts per sample were converted to relative abundance to produce a genus × sample matrix. Sample-wise dissimilarities for functional-gene and taxonomic matrices were calculated with Bray–Curtis distance, and dissimilarities for environmental variables were calculated with Euclidean distance. Mantel tests (Pearson correlation, 999 permutations) were used to assess correlations between biological and environmental distance matrices (same sample set), testing relationships between environmental variables and the abundance of carbon-fixation pathways and their associated microbial communities [43, 44]. Second, the Maximal Information Coefficient (MIC) was estimated to capture the relationships between gene families and environmental factors [45], with MIC ≥ 0.459 and the corresponding *P* ≤ .05 considered as statistically significant (Table S3). The percentage of genes significantly correlated with environmental variables (MIC ≥ 0.459) within the total set of genes involved in the pathway was calculated to estimate the importance of environmental variables for specific pathways. Alpha diversity (Shannon index) was calculated separately for functional genes and microbial communities to investigate diversity at both functional and taxonomic levels(Vegan [46] in R v4.4.1). Genes were first classified into three macro-functional categories: carbon fixation, oxidation processes, and reduction processes. The gene abundance for each category, normalized by TPM, was used to calculate functional alpha diversity. Concurrently, the species abundance table was aggregated at the genus level. Microbial genera associated with each of the three functional categories were extracted, and their relative abundances were used to calculate species alpha diversity. Spearman correlation analysis was performed to estimate the relationships between genes. All statistical analyses were visualized using Chiplot [47] (https://www.chiplot.online/), Tbtools [48], and R packages.

### Molecular ecological network construction and analysis

A molecular ecological co-occurrence network was constructed based on microorganisms and their functional genes involved in carbon fixation and related processes. Three co-occurrence networks were constructed with samples from surface (0–10 cm), middle (50–190 cm), and bottom (390–690 cm), respectively, based on the relative abundances of key genes and associated microbial communities using the Molecular Ecological Network Analysis (MENA) pipeline [49]. These depth groupings were adopted from our previous study on the same sediment cores, in which OTU-level clustering of 16S rRNA gene data identified depth-defined groups [30]. Only taxa and genes present in at least half of the samples were selected to calculate the Spearman correlation coefficient (r), with the minimum threshold (r = 0.95) determined automatically using the RMT method. Network topology parameters were then calculated according to the guidelines provided by the MENA online platform. The constructed networks were visualized by Gephi (0.10.1202301172018, http://gephi.org).

### Metagenome binning and genome annotations

Following the MetaWRAP (v1.3.2) [31] analysis workflow, the contigs obtained from MetaSPADES assembly were binned using the MetaWRAP binning module (parameters: -maxbin2, −concoct, −metabat2). Subsequently, the Bin_refinement and reassemble bins modules were applied to further refine the bins and improve their quality. The quality of the genome bins was evaluated using CheckM (v1.2.2) [50], resulting in 1374 bins with completeness >50% and contamination <10%. Additionally, 336 bins with completeness >85% and contamination <5% were classified as medium-to-high quality. Among them, 205 bins with completeness >90% and contamination <5% were considered high-quality genome bins, hereafter referred to as metagenome-assembled genomes (MAGs, Fig. S3, Table S4). Only medium-to-high quality MAGs were retained for downstream analyses. To identify MAGs with carbon-fixation potential, bins containing all key marker genes required for a given carbon-fixation pathway were considered as possessing carbon-fixation potential. Additionally, pathway completeness for each MAG was assessed using KEGG GhostKOALA [51] annotations combined with automatic pathway reconstruction via KEGGdecoder. The reads per kilobase per million mapped reads (RPKM) of all MAGs across the samples was calculated using CoverM. Additionally, we calculated the content of carbon-fixation genes in carbon-fixation MAGs from surface to deep sediment layers. The taxonomy of MAGs was performed using the GTDB-Tk (v2.4.0) reference data version r220 [52]. Genes were predicted using Prokka (v1.14.6) [53] and annotated with Metabolic and hmmsearch.

### Metabolic reconstruction of representative carbon-fixing MAGs

Representative carbon-fixing groups with relatively high abundance in surface and deep sediment layers were selected for metabolic pathway reconstruction. The 16S rRNA gene sequences were extracted from the MAGs using Barrnap (v0.9) and aligned to previously identified amplicon sequence variants (ASVs) in previous 16S rRNA amplicon sequencing data of 10 SCS sites [30] using BLASTN (v2.5.0+) with a 99% similarity threshold. Matched sequences were retrieved, and 16S-based relative abundances were calculated based on the ASV profiles. Functional annotation was initially performed based on Prokka predictions using GhostKOALA (genus_prokaryotes) [51], and overall pathway reconstruction was conducted via KEGG Mapper [54]. To refine the metabolic reconstruction, additional annotation was carried out using METABOLIC, supplemented with hmmsearch against both the KOfam database [55] and a custom HMM database to improve the resolution of key metabolic functions.

## Results and discussion

### Environmental drivers for carbon fixation and related metabolic processes

Metagenomes of SCS sediments were analyzed for genes coding for carbon fixation and other potential energy metabolisms that sustained it, including the nitrogen cycling, sulfur cycling, and oxidation of H_2_, Fe, Mn, CO, HCOOH and CH_4_. Generally, key genes involved in the CBB, DC/4HB, 3HP/4HB, WL, rTCA, roTCA and rGLY cycle/pathway were detected in these sediment samples. Although several genes involved in 3-HP bi-cycle (*mcl*, *meh*, and *mct*) were identified, the absence of key gene *mcr* suggested the incompleteness of this pathway.

**Figure 1:**
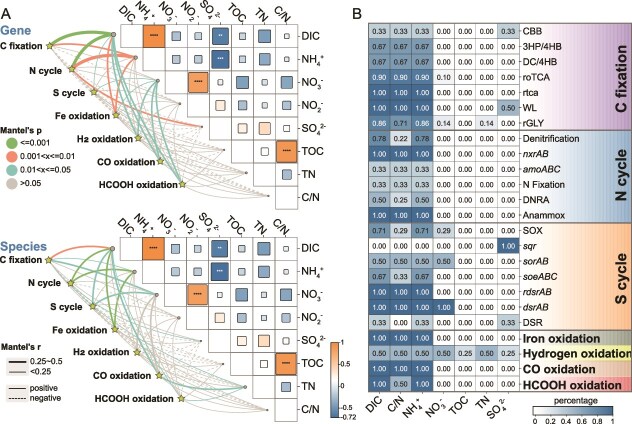
Environmental factors driving microbial carbon fixation and related processes in SCS sediments. (A) Mantel tests based on Bray–Curtis dissimilarity matrices of functional genes and taxonomic profiles and Euclidean distance matrices of environmental variables, evaluating correlations between environmental variation and functional/taxonomic dissimilarity. (B) impact of environmental factor on metabolic pathway indicated by maximal information coefficient (MIC) index. The percentage refers to the proportion of genes involved in specific metabolic pathways significantly driven by environmental factors. DIC: dissolved inorganic carbon. TOC: total organic carbon. TN: total nitrogen. DNRA: dissimilatory nitrate reduction to ammonium. DSR: Dissimilatory sulfate reduction.

Mantel analyses indicated that variation in DIC and NH₄^+^ showed the strongest correlations with variation in gene composition and microbial communities associated with carbon fixation and related processes (Fig. 1A). A significant positive correlation was found between DIC and NH₄^+^, which may be due to the simultaneous production of DIC and NH₄^+^ during the degradation of organic matter in sediments. Both DIC and NH₄^+^ were at relatively low level in 0–90 cm and increased with sediment depth in 100–690 cm [30]. Fermentation oxidation and sulfate reduction are considered the main mechanisms for DIC production in deeper sediments, providing sufficient DIC in deep sediments for these autotrophs [56]. Specifically, the MIC analysis revealed that DIC and NH_4_^+^ explained 66.7%–100% of gene family shifts in the carbon fixation pathways including rTCA, WL, rGLY, roTCA, 3HP/4HB and DC/4HB cycles (Table S3). DIC and NH_4_^+^ also largely influence genes involved in N cycling (anammox, NO₂^−^ oxidation and denitrification), S cycling (*dsrAB* and SOX) and oxidation of Fe, HCOOH and CO (66.7%–100%). As in our previous study, DIC also exhibited a primary influence on the general microbial communities and their diversity [30]. Inconsistent with results of the Mantel test, C/N exhibited a strong correlation in the MIC analysis as DIC and NH_4_^+^ (Fig. 1B), indicating that the availability of organic matter may exert non-liner and indirect influence on inorganic carbon fixation and related energy metabolisms [45]. Additionally, SO_4_^2−^ and NO_3_^−^ also influence the functional and taxonomic diversity in these processes, albeit to a lesser extent (Fig. 1). Collectively, these results indicated that the availability of inorganic carbon and potential electron donor/acceptor such as NH_4_^+^, SO_4_^2−^ and NO_3_^−^ were main drivers for carbon fixation in the SCS sediments. DIC was the most important environmental drivers influencing carbon fixation genes and species which may stimulate microbial chemoautotrophic activity as a biological sink in deep sediments [57].

### General vertical variation trend of carbon fixation and related processes

**Figure 2:**
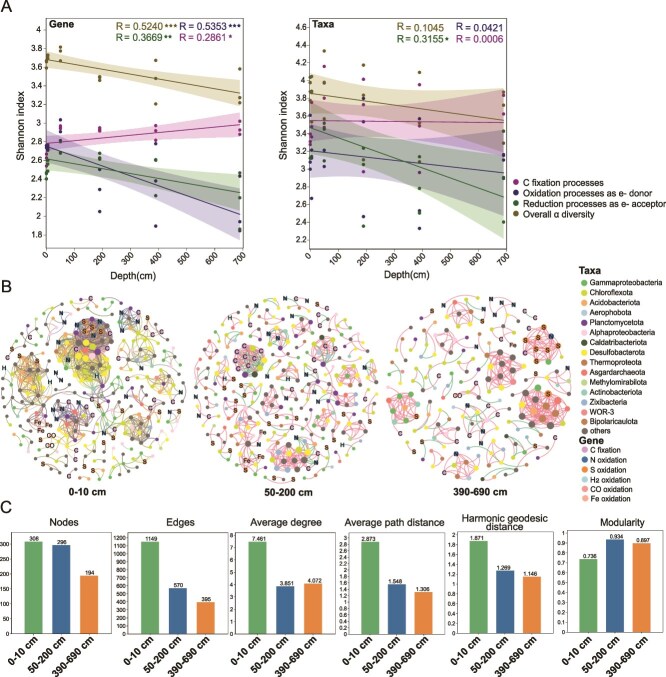
General vertical changes of gene and taxonomic diversity of carbon fixation and related processes. (A) Relationships between gene/taxonomic diversity and sediment depths. Shaded areas represent 95% confidence intervals. R^2^ was obtained by linear regression analysis and P was obtained by Pearson’s correlation analysis. ^*^.01 < *P* < .05); ^**^.001 < *P* < .01; ^***^P < .001. (B) Co-occurrence network of microbial communities and genes related to carbon fixation and related processes across surface, mid-layer, and deep-layer sediments. (C) Overall network topological features of different sediment depths.

The overall α diversity of all functional genes involved in carbon fixation and processes that may provide multiple electron donors/acceptors decreased along sediment depth, while the change of taxonomical diversity was insignificant (Fig. 2A). Notably, the gene diversity in carbon fixation increased with depth, but the diversity of carbon fixation taxa did not show significant differences, indicating that there may be more carbon fixation genes per cell in deep sediments. This phenomenon likely reflects adaptive strategies developed by the deep biosphere under persistent energy limitation and substrate scarcity. Deep-subsurface microorganisms often retain multiple carbon-fixation or autotrophic modules as a form of metabolic redundancy, enabling them to switch among pathways and maintain metabolic robustness under sporadic or limited energy and substrate inputs—a pattern also reported in the Mid-Atlantic Ridge hydrothermal fields [58]. Moreover, because these organisms grow extremely slowly and the energetic cost of genome replication or gene expression is high, maintaining a relatively large and functionally comprehensive genome can be more advantageous over geological timescales. This interpretation is consistent with observations of slow metabolic rates and the long-term stability of genomic repertoires in deep-sediment microbial communities [59]. As for oxidation processes providing electron and energy, the functional diversity was higher in shallow sediments and significantly decreased in deep layers, while the decreasing trend for the taxonomical diversity was statistically insignificant (Fig. 2A). Both the functional and taxonomical diversity of S and N reduction processes acting as electron donors reduced significantly with sediment depths (Fig. 2A).

The co-occurrence networks revealed significant differences in the network structure and topological features at different sediment depths (Fig. 2B). Compared to the middle and deep sediment layers, the surface sediment network was larger and more complex, characterized by more nodes and connections, higher node degrees, higher average path distance and harmonic geodesic distance, and reduced modularity (Fig. 2C). Due to the severe limitations in the availability of electron donors and acceptors for cell respiration and growth [60], the slowed growth rate and metabolism of these microorganisms and the reduction of microbial abundance led to simplified interactions between these autotrophs. This pattern aligns with predictions from consumer–resource models, which indicate that higher energy availability promotes stronger interspecies competition, cross-feeding interactions, and niche differentiation, thereby supporting greater species diversity and more complex network structures [61]. In the present study, surface sediments contained higher concentrations of nutrients such as nitrate, likely contributing to the observed increased network complexity [30]. However, the transitivity and connectedness of these networks increased from surface to deep sediments [62], indicating that although the nodes and links reduced, the carbon fixation network tended to be tighter and more cohesive in deep sediments. Additionally, more positive correlations were found in the carbon fixation network of deep sediments (90.13%) compared to those of the surface and middle layers (85.64% and 82.46%, respectively). Microbial species with highest degree (41) in the network of surface sediments included those from Desulfobacterota (4 species), Planctomycetota, Alphaproteobacteria, Bacteroidota and Asgardarchaeota, while in the deep sediments, species with highest degree (14) were mainly archaea from Thermoproteota, Asgardarchaeota and Nanoarchaeota, and one bacterial species from Acidobacteriota (Table S5).

### Vertical changes of gene profiles in carbon fixation and related processes

**Figure 3:**
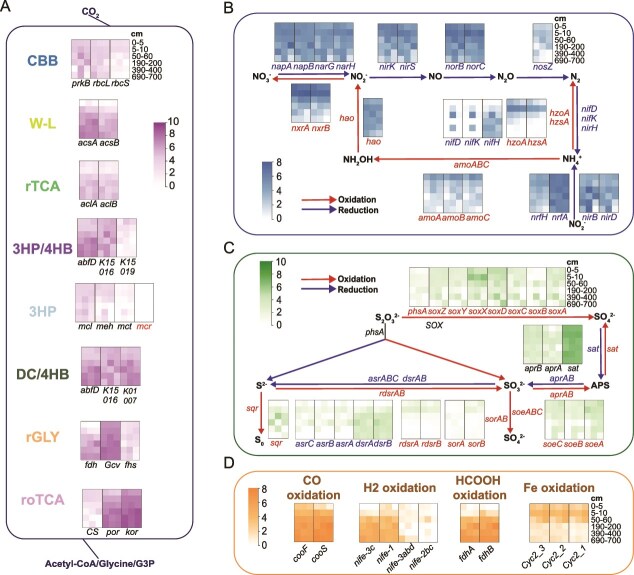
The relative abundance of key genes involved in carbon fixation and related processes. (A) Genes involved in carbon fixation pathways. (B) genes involved in oxidation and reduction processes of nitrogen cycling. (C) genes involved in oxidation and reduction processes of sulfur cycling. (D) depth-dependent variations in the abundance of key genes related to other oxidation pathways. The relative abundance was calculated as TPM.

To further elucidate the detailed variation patterns of different genes and pathways with sediment depth, we analyzed the relative abundance of key functional genes in different carbon fixation pathways and related metabolic processes, and many of these genes exhibited a clear depth-dependent distribution pattern (Fig. 3).

(1) **Carbon fixation**

The distribution of marker genes in carbon fixation pathways presented a clear stratification between oxic (0–10 cm) and anoxic sediment (50–690 cm). Genes encoding the large subunits of RuBisCO (*rbcL*) and phosphoribulokinase (*prkB*) in the CBB cycle were ubiquitous throughout all depths, whereas another key gene encoding the small subunits of RuBisCO (*rbcS*) mainly existed in 0–5 cm, indicating that the CBB cycle was dominant in the oxic sediments. The prevalence of CBB cycle in the surface sediments have been revealed in diverse environments such as estuarine, gulf and coastal sediments, mainly performed by γ-Proteobacteria [12, 63–65]. In contrast, the abundance of *acsA* and *acsB*, which encode anaerobic carbon monoxide dehydrogenase and acetyl-CoA synthase in the WL pathway, increased with sediment depth and reached highest in 190–690 cm. Similar to other anoxic environments [66–68], the WL pathway acted as the most energy-efficient carbon fixation mechanism [69] and was prevalent in the dominant microbial groups in the deep anoxic sediments. Another anaerobic carbon fixation pathway, the rTCA cycle was also enriched in 50–690 cm, but the abundance of its key genes was lower than those of the WL cycle. For rGLY and roTCA pathways, the abundance of their representative genes showed inconsistent variation with sediment depths. However, considering the distribution of key gene *fhs* and *CS* (citrate synthase) in the rGLY and roTCA pathways, respectively, these two pathways tended to be more enriched in the upper sediments. Additionally, lack of partial key genes of the 3HP (*mcr*) indicated the incompleteness of the pathway in the SCS sediments, although genes encoding for 4-hydroxybutyrate dehydratase (*abfD*) and 3-hydroxyacyl-CoA dehydrogenase (*K15016*) shared by 3HP/4HB and DC/4HB pathways increased with sediment depth. Notably, another key gene in the 3HP/4HB pathway, 3-hydroxypropionyl-coenzyme A dehydratase (*K15019*), exhibited relatively low abundance and decreased with depth, suggesting that this pathway was more abundant in surface sediments. In contrast, key genes in the DC/4HB pathway consistently enriched in sediments below 50 cm (Fig. 3A).

(2) **Oxidation processes as e**^**−**^  **donor**

Oxidation of nitrogen compounds is an important energy source and electron donor for carbon fixation [30]. It is estimated that denitrification in continental shelf sediments accounts for 44% of global denitrification [70]. In the SCS sediment, such oxidation processes were highly enriched in the shallow sediments (0–50 cm). For anammox and ammonia oxidation, the abundance of *hzoA*, *hzsA* and *amoABC* was highest in 0–5 cm; *hzoA* and *hzsA* were nearly absent in the deep sediments. The abundance of the *nxrAB* gene responsible for oxidizing nitrite to nitrate was extremely high in 0–5 cm and decreased with depths. The nitrite-oxidizing bacteria (NOB) and ammonia-oxidizing archaea (AOA) in the surface sediment together catalyze the oxidation of ammonium to nitrate and drive the carbon fixation processes. These results suggested that oxidation of nitrogen compounds, especially nitrite oxidation, could be the dominant driver for carbon fixation in shallow sediments. Notably, limited oxygen and abundant nitrite at 5–10 cm resulted in the enrichment of genes associated with nitrite oxidation and reduction, including those for Anammox. In contrast, *nifH* gene indicative for nitrogen fixation were abundant in deep sediments (Fig. 3B).

Generally, the relative abundance of genes involved in sulfur cycling that may be coupled with carbon fixation was lower than those in nitrogen cycling, and this differs from existing reports suggesting that sulfur oxidation serves as the predominant chemolithotrophic process in coastal sediments [12, 14]. For sulphide oxidation to elemental sulfur, the *sqr* gene showed higher abundance in 5–190 cm. The SOX system (*soxXABCDYZ*) responsible for the thiosulfate oxidation to sulfate, generally declined along sediment depth. However, the essential gene *soxB* was nearly absent in surface sediments, suggesting that thiosulfate oxidation mainly occurs in the middle and deep sediment layer. Genes responsible for sulfite oxidation to sulfate (*soeABC* and *sorAB*) showed higher abundance in sediments above 50 cm (Fig. 3C). The *rdsrAB* genes responsible for sulfide oxidation existed primarily in the shallow sediments. Other sulfur oxidation genes, such as *fccB* and *sor*, were not detected in these sediment samples (Fig. 3C). Therefore, the oxidation of sulfur compound that may fuel carbon fixation mainly took place in the surface and middle sediments within the upper 190 cm.

For other potential energy sources for carbon fixation, genes responsible for H_2_ oxidation ([NiFe]-hydrogenase), HCOOH oxidation (*fdhAB*), and CO oxidation (*cooSF*), exhibited a relative abundance that generally increased with sediment depth (Fig. 3D). This suggests that H_2_/HCOOH/CO oxidation may serve as the primary electron donors and energy sources for carbon fixation in middle and deep sediments. In contrast, genes related to Fe oxidation (*cyc2*) showed relatively low abundance and decreased with sediment depth, primarily found in surface and middle sediments. These findings suggest that while H_2_/HCOOH/CO oxidation may play a more significant role in deep sediments, iron oxidation is more prominent in surface sediments, likely serving as an alternative electron donor for carbon fixation and other biogeochemical processes within the microbial community.

(3) **Reduction processes as e**^**−**^  **acceptor**

For the reduction processes in the nitrogen cycling as an electron sink of carbon fixation, the abundance of genes involved in the denitrification process from NO_3_^−^ to N_2_ (*napAB*/*narGH*, *nirKS*, *norBC,* and *nosZ*) were detected with high relative abundances but with a decreased trend along the sediment depth. As for dissimilatory nitrate reduction to ammonium (DNRA), the abundance of the *nrfAH* decreased with increasing sediment depth, and *nirBD* did not show significant differences between depths. Due to the high NO_3_^−^ and NO_2_^−^ content [30] and potentially abundant intermediate nitrogen compounds in sediments above 50 cm, nitrification, ammonia oxidation and denitrification processes were highly active in these depths supporting carbon fixation process (Fig. 3B). As for the sulfur reduction processes, *asrABC* responsible for sulfite reduction to sulfide decreased with sediment depth. Genes involved in dissimilatory sulfate reduction (*sat* and *aprAB*) showed no discernable variations with sediment depth. The *dsrAB* for sulfite reduction were primarily found in the middle and deep sediment (Fig. 3C).

The correlations between oxidation and reduction genes were also examined to identify the potential coupled redox processes (Fig. S4). Notably, genes involved in denitrification (e.g. *narGH*, *nrfAH*, *norBC*, *nirBD*, *nirKS,* and *nifDKH*) showed a significant positive correlation (*P <* .001) with sulfur oxidation genes (e.g. *soxXABCDY*, *rdsrAB*, *sorAB*, *soeABC,* and *sqr*), as previously reported in the marine sediment [71], and these processes occurred in all depths with slightly higher abundance of related genes in above 50–60 cm. Similarly, nitrite oxidation genes (*nxrAB*) and Fe oxidation genes (*cyc2*) were significantly positively correlated with several denitrification genes such as *narH*, *nrfH*, *nirK* and *norC*. While in the deep sediments, *dsrAB* genes catalyzing sulfate reduction showed a significant positive correlation with H_2_/CO/HCOOH oxidation genes (*P* < .001). These results indicated distinct coupling e^−^ donors and acceptors along sediment depths that support carbon fixation.

### Genomic capacity for carbon fixation and coupling processes

**Figure 4:**
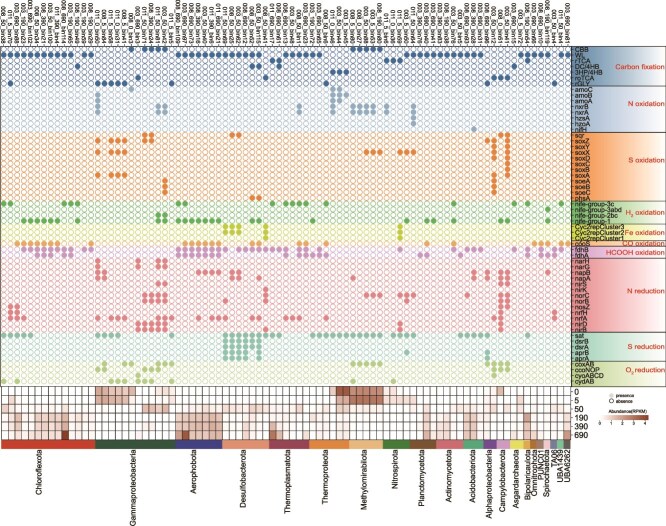
Carbon fixation and associated redox processes of carbon-fixing MAGs and their abundance across sediment depths. In the upper panel, circle colors indicate the presence or absence of the corresponding functional genes. In the lower heatmap, the relative abundance (RPKM) was calculated as an average of three samples from the same depth.

A total of 336 MAGs were retrieved from the SCS sediment (completeness >85%, contamination <5%, Fig. S3), including 299 bacterial MAGs and 37 archaeal MAGs (Fig. S5). Among them, 85 MAGs were predicted to possess carbon fixation potential (Fig. 4, Table S6). Furthermore, by comparing MAGs recovered from different sediment depths, we quantified the number of key carbon-fixation marker genes per MAG, and the results were consistent with the aforementioned α-diversity patterns: MAGs from surface sediments contained significantly fewer carbon-fixation marker genes than those from mid-to-deep layers, further supporting that deep-sediment carbon-fixing microbes tend to harbor a greater diversity of carbon-fixation genes within a single genome (Fig. S6).

**Figure 5:**
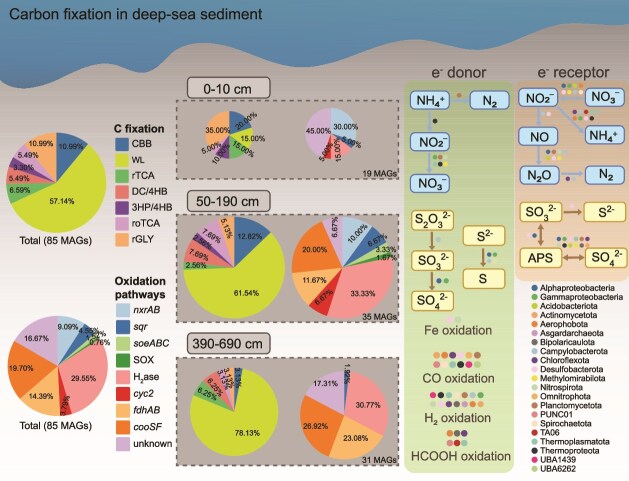
Conceptual model of microbial carbon fixation and redox processes and their coupling mechanisms in the SCS sediments. The pie charts represent the relative proportions of MAGs associated with different carbon fixation pathways and oxidation processes. The size of each pie chart reflects the number of MAGs at each depth. Colored dots indicate microbial taxa involved in each pathway.

The WL pathway was widely annotated across anaerobic MAGs distributed in deep sediments, belonging to Desulfobacterota, Chloroflexota, Aerophobota, and Thermoplasmatota (52 out of 85, Fig. 5). Further analysis of the WL pathways in these MAGs revealed that the vast majority (48 of 52) harbored complete carbonyl branches, a core component of WL pathway. Among these 48 MAGs, two Actinomycetota MAGs contained complete tetrahydrofolate (THF)-based methyl branches. An additional four MAGs belonging to Chloroflexota and Aerophobota, although lacking the known key enzyme FDH, contain sequences annotated as FDH-like based on gene prediction. Similar observations have been reported for carbon-fixing microorganisms in blue hole environments (Table S7) [66]. However, the functional identity of these sequences requires further verification through phylogenetic or structural analyses. Notably, three Bathyarchaeia MAGs and one Asgardarchaeota MAG contained only the H_4_MPT-based methyl branch without the carbonyl branch. However, these archaea possess an archaeal-specific acetyl-CoA synthesis pathway, in which *cdhAB* (carbon monoxide dehydrogenase) can functionally substitute for the carbonyl branch of the WL pathway by reducing CO_2_ to CO. This CO can then combine with the methyl branch intermediate to form acetyl-CoA, thereby completing a functionally intact WL carbon fixation pathway (Fig. S7, Table S7). Previous studies have shown that archaea frequently employ *cdhAB* to catalyze CO₂ reduction to CO, and that the *cdhB* subunit is archaeal-specific, supporting this alternative carbonyl-branch mechanism [72]. Additionally, Bathyarchaeia from lake sediments have been reported to possess genomic potential for the WL pathway [73], consistent with our observations.

The CBB pathway was predicted in 10 MAGs affiliated with Gammaproteobacteria and Methylomirabilia from the 0–10 cm surface sediments, all of which exhibited 100% pathway completeness (Fig. S8A, Table S8, Table S9). The high abundance of Methylomirabilia MAGs indicated that they could be an important autotrophic group in the surface sediments (Fig. 4). Additionally, key genes of the 3HP/4HB cycle were exclusively found in ammonia-oxidizing Nitrososphaeria archaea and exhibited exceptionally high abundance in surface sediments (Fig. 4, Fig. S8A). The rTCA pathway was predicted in three MAGs from Nitrospirota distributed above 10 cm, and in three MAGs from Bipolaricaulota and Thermoplasmatota (DHVEG-1) in deep sediments. Key genes of the DC/4HB pathway were annotated in MAGs from deep sediments, including three archaeal (two Thermoplasmatota and one Asgardarchaeota) and two bacterial (Desulfobacterota and Chloroflexota) MAGs. Previous studies suggested that the DC/4HB pathway is primarily found in archaea from extreme environments, such as high temperatures and low oxygen conditions [67, 74]. However, our findings suggest that DC/4HB pathway may also exist in certain bacteria such as Chloroflexota and Desulfobacterota, though its activity requires further validation. Except the Asgardarchaeota MAG, the DC/4HB pathway coexisted with other carbon fixation pathways (WL and rTCA) in other four MAGs (Fig. 4). Genes for rGLY pathway were identified in five Acidiferrobacterales MAGs of Gammaproteobacteria in the surface sediments, as well as in five other MAGs (such as those from TA06 and Chloroflexota) from deeper sediments. Additionally, the roTCA pathway was encoded by five MAGs from Gammaproteobacteria, Alphaproteobacteria, Desulfobacterota, and Campylobacterota, with no significant variation pattern with depth (Fig. 4, Fig. S8A).

We found that CO, HCOOH and H_2_ oxidation served as crucial energy sources for carbon fixation in deep sediments (Fig. 5, Fig. S8B). The majority of Chloroflexota bacteria possessing the WL pathway also harbored genes for CO and HCOOH oxidation and some contained H_2_ oxidation genes, while genes related to N and S reduction were rare (Fig. 4). A single Dehalococcoidia MAG (008_190_bin49) differed from other Chloroflexota MAGs and may utilize a DC/4HB pathway driven by H_2_ oxidation (Fig. 4). Similarly, deep-layer Aerophobota MAGs supported their WL pathway through CO and HCOOH oxidation, and all these MAGs also possessed H_2_ oxidation gene. Additionally, H_2_/CO/HCOOH oxidation also supported the WL pathway in Thermoplasmatota, Planctomycetota, Actinobacteriota and Acidobacteriota. For sulfate-reducing Desulfobacterota MAGs, carbon fixation pathways are more diverse, and many of them performed iron oxidation alongside CO and HCOOH oxidation (Fig. 4).

In 0–50 cm sediment layers, diverse carbon fixation pathways (CBB, rGly, and rTCA) in Gammaproteobacteria are primarily supported by sulfur oxidation, while various N reduction processes providing electron acceptors. The CBB cycling in the highly abundant Methylomirabilota in the surface sediments were driven mainly by nitrite oxidation. Meanwhile, the 3HP/4HB pathway in Nitrososphaeria is powered by their characteristic ammonia oxidation process (Fig. 4). To summarize, nitrite and sulfur oxidation provided primary energy for carbon fixation in the shallow sediments, while H_2_/CO/HCOOH oxidation were the main energy sources in deep sediments (Fig. 5, Fig. S8B). However, 21 MAGs lacked any of the oxidation pathways mentioned above, leaving their energy sources unknown (Fig. 4). Several factors may explain this observation. First, incomplete assembly of the MAGs may have resulted in the loss of key metabolic genes. Second, these microorganisms may inhabit dense microbial communities and acquire energy indirectly through the exchange of metabolites with other microbes [75]. Finally, some organisms may employ a mixotrophic strategy, simultaneously performing carbon fixation while utilizing simple organic compounds from the environment to meet both energy and carbon requirements [76]. In our dataset, many carbon-fixing MAGs also encode heterotrophic pathways, which further supports the idea that MAGs lacking canonical oxidative routes are more likely to rely on mixotrophy (Fig. S9).

Four types of terminal oxidases responsible for oxygen reduction were identified in several MAGs from 0 to 10 cm sediment. Apart from oxygen as an electron acceptor, MAGs performed N reduction processes were predominantly distributed in the upper 10 cm of sediments, with some exhibiting high abundance at 50 cm depth. Although partial N reduction genes were detected in certain MAGs from deeper layers, most were incomplete. Sulfate reduction has been considered as a dominant process in deep sediment, as suggested by the distribution of *dsrAB*. However, *dsrAB* genes were missing from most carbon fixation MAGs expect those from Desulfobacterota. Additionally, while the sulfate activation gene *sat* was widely detected among carbon-fixing MAGs, the downstream reduction gene *aprAB* was found in only seven MAGs. This suggests that APS, once generated, may primarily enter assimilatory sulfate reduction pathways rather than be used in energy metabolism. In deep sediment, the absence of electron acceptors in these MAGs may be attributed to that two CO_2_ molecules as terminal electron acceptors in their WL pathway, requiring no external inorganic electron acceptors [77–79]. Previous studies have consistently reported this phenomenon. Specifically, the WL pathway, first elucidated mainly in the thermophilic acetogen *Moorella thermoacetica* (formerly Clostridium thermoaceticum), is named after its discoverers [77]. The pathway contains two branches, each responsible for reducing one molecule of CO₂. In the carbonyl branch, CO₂ is reduced to enzyme-bound CO by the CO dehydrogenase/acetyl-CoA synthase (CODH/ACS). During this process, CO₂ functions as the terminal electron acceptor of an anaerobic respiration [80]. Consistent with previous findings [79, 81], most of our WL-containing MAGs also harbor key genes associated with acetogenesis, suggesting that these MAGs possess the potential to perform WL-based autotrophic acetogenesis without relying on additional inorganic electron acceptors. Additionally, no complete *mtrABC* genes were detected in these MAGs, suggesting that manganese and iron reduction was not a key process providing electron acceptors in these sediments.

Furthermore, analysis of carbohydrate-active enzymes (CAZymes) in the MAGs revealed many of them can also utilize various organic carbon compounds; the amounts and types of CAZymes varied between MAGs of different carbon fixation pathways and oxidation pathways (Table S10). In 10 MAGs with the complete CBB cycle, four Gammaproteobacteria MAGs also possessed either complete tricarboxylic acid (TCA) cycle or complete Entner–Doudoroff (ED) pathway, suggesting their mixotrophic lifestyle (Table S8, Table S9). While for MAGs with WL pathway, the TCA cycle and glycolytic pathways were generally incomplete, but they possessed more genes involved in complex carbon degradation. Additionally, three Gammaproteobacteria MAGs containing the complete rGly pathway exhibited high completeness of glycolysis and TCA cycles and showed potential to metabolize formate and acetate. These mixotrophic carbon-fixing microorganisms not only fix CO₂ via autotrophic pathways but also utilize a range of organic substrates, conferring a distinct ecological advantage in the nutrient-variable deep-sea sediment environment (Fig. S9). The mixotrophic lifestyle of the MAGs, combining autotrophic carbon fixation with heterotrophic utilization of organic substrates, likely confers a survival advantage in energy- and substrate-limited deep-sea sediments. Such versatility allows microorganisms to switch between or simultaneously employ autotrophy and heterotrophy depending on substrate availability, maintaining metabolic flexibility and resilience under oligotrophic conditions [82, 83]. Similar strategies have been observed in deep-sea sediment archaea and bacteria, where mixotrophy supports persistent energy acquisition despite intermittent nutrient input [66, 84].

### Metabolic characterization of dominant carbon-fixing microorganisms in sediments

**Figure 6:**
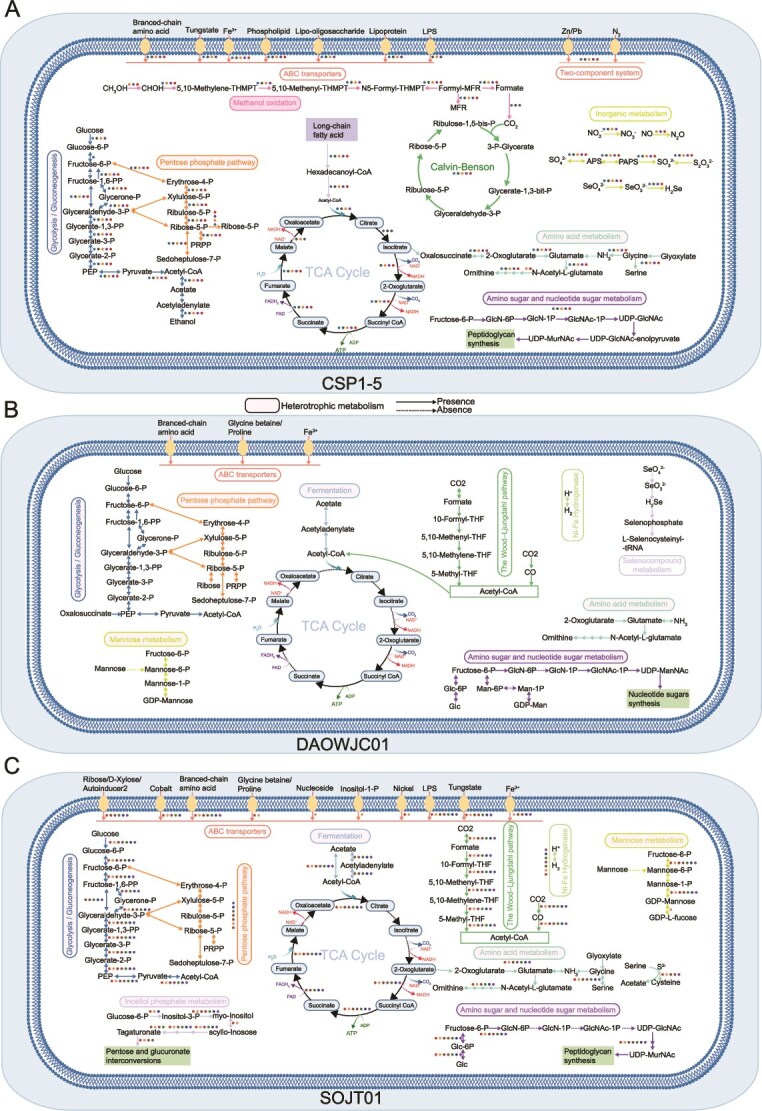
Metabolic modeling of dominant carbon-fixing microbial groups in deep-sea sediments. (A) CSP1–5 (Methylomirabilota); (B) DAOWJC01 (Chloroflexota); (C) SOJT01 (Aerophobota). Rectangles in different colors represent distinct metabolic modules, with filled rectangles indicating reactions associated with heterotrophic metabolism.

We further investigated the detailed metabolic processes of representative carbon-fixing MAGs with high abundance in deep-sea sediments (Fig. S10). In sediments above 10 cm, five Methylomirabilota CSP1–5 MAGs possessing a complete CBB cycle driven by nitrite oxidation were highly abundant, especially in the 5–10 cm layer with high NO_2_^−^ concentrations [30] (Fig. S10). Their carbon fixation process was driven primarily by nitrite oxidation, with sulfur compounds serving as the electron acceptor. The near-complete TCA cycle, glycolysis/gluconeogenesis and pentose phosphate pathway (PPP) indicated these Methylomirabilota MAGs were mixotrophic (Table S8, Table S9). Consistent with previous studies [85], two CSP1–5 MAGs identified possessed complete pathway to oxide formaldehyde to formate, and the resulting formate can be subsequently oxidized to CO₂ by formate dehydrogenase and assimilated via the CBB cycle [85]. The methanol oxidation pathways in other three CSP1–5 MAGs were incomplete. All CSP1–5 MAGs in this study showed the potential for acetate utilization, indicating a broader energy source spectrum than previously reported (Table S11, Fig. 6A). Four CSP1–5 MAGs harbored the arsenite methyltransferase gene (*K07755*), which catalyzes the methylation of arsenite and plays a critical role in arsenic metabolism and detoxification. Additionally, these MAGs also showed potential for selenate reduction and fatty acid utilization (Table S11, Fig. 6A).

One Dehalococcoidia MAG in Chloroflexota (008–690-bin114) exhibited an extremely high abundance at 690 cm (Fig. S10). This MAG harbored a complete WL pathway, including both the tetrahydrofolate (THF)-dependent methyl branch and the carbonyl branch. H_2_ oxidation is likely the main energy source driving the WL pathway, while selenite reduction may serve as a unique electron acceptor [86] that was not identified in other Dehalococcoidia MAGs (Table S11). Additionally, complete glycolysis/gluconeogenesis and PPP were found in this MAG, whereas the incomplete TCA cycle may function primarily to supply metabolic intermediates (Fig. 6B). Currently, no deep-sea sediment Dehalococcoidia has yet been cultured and isolated, and although Dehalococcoidia species were known for their obligate organohalide respiration, no reductive dehalogenase (Rdase) genes were found in our Dehalococcoidia MAG as reported for other deep-sea sediment Dehalococcoidia genomes [87–89] (Table S11). Compared to Dehalococcoidia MAGs harboring Rdase genes, those from marine sediments carried more diverse genes related to sulfur metabolism (Table S11). In contrast to previous genomes lacking Rdase, which often utilize dissimilatory dimethyl sulfoxide (DMSO) reductases [87] or sulfite reductases [90] as terminal electron acceptors, our MAG harbors neither of these enzymes. Instead, the presence of selenite reductase provides the only genomic evidence for its potential terminal electron acceptor.

Seven Aerophobota MAGs also showed high abundance in deep sediments below 190 cm (Fig. S10), containing complete WL pathway supported by hydrogen oxidation (Table S11). Regarding carbohydrate metabolism, these Aerophobota MAGs contained nearly complete glycolysis/gluconeogenesis pathways and complete PPP, although their TCA cycles were incomplete (Fig. 6C). The presence of diverse transmembrane sugar transporter genes further indicates their capacity to utilize sediment-preserved carbohydrates. In addition, they also exhibited the potential for acetate fermentation (Fig. 6C). Formate oxidation genes were identified in all our Aerophobota MAGs, as in a previously reported Aerophobota MAG from the SCS sediment [91], but not in those MAGs from Thuwal cold seeps in the Red Sea [92] and the Eastern Gulf of Mexico sediment [93]. The above results indicate that the representative carbon-fixing MAGs in the deep-sea sediments exhibit certain differences compared to their counterparts in other environments.

## Conclusions

Our in-depth investigation of carbon-fixing microbial communities in the SCS deep-sea sediments revealed vertical stratification of diverse carbon fixation pathways, linked to the availability of oxygen, DIC and electron donors/receptors. The CBB cycle and the rGLY pathway dominate in the surface sediment, supported mainly by nitrite oxidation and denitrification processes. In contrast, the WL pathway driven by hydrogen and carbon monoxide oxidation were dominant in the anoxic deep sediments. The shift in carbon fixation pathways and energy sources in deep-sea sediments were distinct from those observed in coastal sediments, and the representative carbon-fixing groups from Methylomirabilota, Dehalococcoidia and Aerophobetes exhibited different metabolic features compared to those from other environments. These results underscore the role of deep-sea sediment microbes in global carbon sequestration, offering insights into their resilience under energy limitation and their capacity to drive biogeochemical cycles.

## Supplementary Material

supplementary_figures_ycaf242

supplementary_tables_ycaf242

## Data Availability

Raw metagenomic sequencing reads generated in this study have been deposited in the NCBI Sequence Read Archive (SRA) under BioProject accession PRJNA1300796. Processed data files are available from the corresponding author upon reasonable request.

## References

[1] Tyler PA . Ecosystems of the Deep Oceans. Amsterdam: Elsevier, 2003.

[2] Zhang C, Peng Y, Liu X. et al. Deep-sea microbial genetic resources: new frontiers for bioprospecting. *Trends Microbiol* 2024;32:321–4. 10.1016/j.tim.2024.01.002 38290879

[3] Parkes RJ, Cragg BA, Wellsbury P. Recent studies on bacterial populations and processes in subseafloor sediments: a review. *Hydrogeol J* 2000;8:11–28. 10.1007/PL00010971

[4] Zinger L, Amaral-Zettler LA, Fuhrman JA. et al. Global patterns of bacterial beta-diversity in seafloor and seawater ecosystems. *PLoS One* 2011;6:e24570. 10.1371/journal.pone.0024570 21931760 PMC3169623

[5] Mason OU, Di Meo-Savoie CA, Van Nostrand JD. et al. Prokaryotic diversity, distribution, and insights into their role in biogeochemical cycling in marine basalts. *ISME J* 2009;3:231–42. 10.1038/ismej.2008.92 18843298

[6] Berner RA . Burial of organic carbon and pyrite sulfur in the modern ocean; its geochemical and environmental significance. *Am J Sci* 1982;282:451–73. 10.2475/ajs.282.4.451

[7] Nealson KH . Sediment bacteria: who’s there, what are they doing, and what’s new? *Annu Rev Earth Planet Sci* 1997;25:403–34. 10.1146/annurev.earth.25.1.403 11540735

[8] Hedges JI, Keil RG. Sedimentary organic matter preservation: an assessment and speculative synthesis. *Mar Chem* 1995;49:81–115. 10.1016/0304-4203(95)00008-F

[9] Burdige DJ . Preservation of organic matter in marine sediments: controls, mechanisms, and an imbalance in sediment organic carbon budgets? *Chem Rev* 2007;107:467–85. 10.1021/cr050347q 17249736

[10] Parkes RJ, Cragg BA, Getliff JM. et al. A quantitative study of microbial decomposition of biopolymers in recent sediments from the Peru margin. *Mar Geol* 1993;113:55–66. 10.1016/0025-3227(93)90149-P

[11] Middelburg JJ . Chemoautotrophy in the ocean. *Geophys Res Lett* 2011;38:L24604. 10.1029/2011GL049725

[12] Dyksma S, Bischof K, Fuchs BM. et al. Ubiquitous Gammaproteobacteria dominate dark carbon fixation in coastal sediments. *ISME J* 2016;10:1939–53. 10.1038/ISMEJ.2015.257 26872043 PMC4872838

[13] Vasquez-Cardenas D, Meysman FJR, Boschker HTS. A cross - system comparison of dark carbon fixation in coastal sediments. *Glob Biogeochem Cycles* 2020;34:e2019GB006298. 10.1029/2019GB006298 PMC7375125 32713991

[14] Liu B, Hou L, Zheng Y. et al. Dark carbon fixation in intertidal sediments: controlling factors and driving microorganisms. *Water Res* 2022;216:118381. 10.1016/j.watres.2022.118381 35381430

[15] Boschker HTS, Vasquez-Cardenas D, Bolhuis H. et al. Chemoautotrophic carbon fixation rates and active bacterial communities in intertidal marine sediments. *PLoS One* 2014;9:e101443. 10.1371/journal.pone.0101443 25003508 PMC4086895

[16] Molari M, Manini E, Dell’Anno A. Dark inorganic carbon fixation sustains the functioning of benthic deep-sea ecosystems. *Glob Biogeochem Cycles* 2013;27:212–21. 10.1002/gbc.20030

[17] Correa SS, Schultz J, Lauersen KJ. et al. Natural carbon fixation and advances in synthetic engineering for redesigning and creating new fixation pathways. *J Adv Res* 2023;47:75–92. 10.1016/j.jare.2022.07.011 35918056 PMC10173188

[18] Becerra A, Rivas M, Garcia-Ferris C. et al. A phylogenetic approach to the early evolution of autotrophy: the case of the reverse TCA and the reductive acetyl-CoA pathways. *Int Microbiol* 2014;17:91–7. 10.2436/20.1501.01.211 26418853

[19] Garritano AN, Song W, Thomas T. Carbon fixation pathways across the bacterial and archaeal tree of life. *PNAS Nexus* 2022;1:pgac226. 10.1093/pnasnexus/pgac226 36712370 PMC9802188

[20] Lennarz WJ, Lane MD. *Encyclopedia of Biological Chemistry*. Cambridge, Massachusetts: Academic Press, 2013.

[21] Wuchter C, Abbas B, Coolen MJL. et al. Archaeal nitrification in the ocean. *Proc Natl Acad Sci USA* 2006;103:12317–22. 10.1073/pnas.0600756103 16894176 PMC1533803

[22] Herndl GJ, Reinthaler T, Teira E. et al. Contribution of archaea to total prokaryotic production in the deep Atlantic Ocean. *Appl Environ Microbiol* 2005;71:2303–9. 10.1128/AEM.71.5.2303-2309.2005 15870315 PMC1087563

[23] Reinthaler T, van Aken HM, Herndl GJ. Major contribution of autotrophy to microbial carbon cycling in the deep North Atlantic’s interior. *DEEP-SEA Res PART II-Top Stud Oceanogr* 2010;57:1572–80. 10.1016/j.dsr2.2010.02.023

[24] Klatt JM, Polerecky L. Assessment of the stoichiometry and efficiency of CO2 fixation coupled to reduced sulfur oxidation. *Front Microbiol* 2015;6:484. 10.3389/fmicb.2015.00484 26052315 PMC4440400

[25] Bowles MW, Mogollon JM, Kasten S. et al. Global rates of marine sulfate reduction and implications for sub-sea-floor metabolic activities. *Science* 2014;344:889–91. 10.1126/science.1249213 24812207

[26] Lappan R, Shelley G, Islam ZF. et al. Molecular hydrogen in seawater supports growth of diverse marine bacteria. *Nat Microbiol* 2023;8:581–95. 10.1038/s41564-023-01322-0 36747116 PMC10305171

[27] Cordero PRF, Bayly K, Leung PM. et al. Atmospheric carbon monoxide oxidation is a widespread mechanism supporting microbial survival. *ISME J* 2019;13:2868–81. 10.1038/s41396-019-0479-8 31358912 PMC6794299

[28] Dong L, Wang X, Tong H. et al. Distribution and correlation of iron oxidizers and carbon-fixing microbial communities in natural wetlands. *Sci Total Environ* 2024;912:168719. 10.1016/j.scitotenv.2023.168719 38040374

[29] Zheng Y, Wang H, Liu Y. et al. Electrochemically coupled CH4 and CO2 consumption driven by microbial processes. *Nat Commun* 2024;15:3097. 10.1038/s41467-024-47445-8 38600111 PMC11006836

[30] Zhang Y, Yao P, Sun C. et al. Vertical diversity and association pattern of total, abundant and rare microbial communities in deep-sea sediments. *Mol Ecol* 2021;30:2800–16. 10.1111/mec.15937 33960545 PMC8251536

[31] Uritskiy GV, DiRuggiero J, Taylor J. MetaWRAP-a flexible pipeline for genome-resolved metagenomic data analysis. *MICROBIOME* 2018;6:158. 10.1186/s40168-018-0541-1 30219103 PMC6138922

[32] Nurk S, Meleshko D, Korobeynikov A. et al. metaSPAdes: a new versatile metagenomic assembler. *Genome Res* 2017;27:824–34. 10.1101/gr.213959.116 28298430 PMC5411777

[33] Hyatt D, Chen G-L, LoCascio PF. et al. Prodigal: prokaryotic gene recognition and translation initiation site identification. *BMC Bioinformatics* 2010;11:119. 10.1186/1471-2105-11-119 20211023 PMC2848648

[34] Li W, Godzik A. Cd-hit: a fast program for clustering and comparing large sets of protein or nucleotide sequences. *Bioinformatics* 2006;22:1658–9. 10.1093/bioinformatics/btl158 16731699

[35] Li H, Durbin R. Fast and accurate short read alignment with burrows-wheeler transform. *Bioinformatics* 2009;25:1754–60. 10.1093/bioinformatics/btp324 19451168 PMC2705234

[36] Aroney STN, Newell RJP, Nissen JN. et al. CoverM: read alignment statistics for metagenomics. *Bioinforma Oxf Engl* 2025;41:btaf147. 10.1093/bioinformatics/btaf147 PMC11993303 40193404

[37] Danecek P, Bonfield JK, Liddle J. et al. Twelve years of SAMtools and BCFtools. *GIGASCIENCE* 2021;10:giab008. 10.1093/gigascience/giab008 33590861 PMC7931819

[38] Zhou Z, Tran PQ, Breister AM. et al. METABOLIC: high-throughput profiling of microbial genomes for functional traits, metabolism, biogeochemistry, and community-scale functional networks. *MICROBIOME* 2022;10:33. 10.1186/s40168-021-01213-8 35172890 PMC8851854

[39] Mueller AL, Kjeldsen KU, Rattei T. et al. Phylogenetic and environmental diversity of DsrAB-type dissimilatory (bi) sulfite reductases. *ISME J* 2015;9:1152–65. 10.1038/ismej.2014.208 25343514 PMC4351914

[40] Shen W, Le S, Li Y. et al. SeqKit: a cross-platform and ultrafast toolkit for FASTA/Q file manipulation. *PLoS One* 2016;11:e0163962. 10.1371/journal.pone.0163962 27706213 PMC5051824

[41] Buchfink B, Xie C, Huson DH. Fast and sensitive protein alignment using DIAMOND. *Nat Methods* 2015;12:59–60. 10.1038/nmeth.3176 25402007

[42] Huson DH, Auch AF, Qi J. et al. MEGAN analysis of metagenomic data. *Genome Res* 2007;17:377–86. 10.1101/gr.5969107 17255551 PMC1800929

[43] Qian L, Yu X, Gu H. et al. Vertically stratified methane, nitrogen and Sulphur cycling and coupling mechanisms in mangrove sediment microbiomes. *Microbiome* 2023;11:71. 10.1186/s40168-023-01501-5 37020239 PMC10074775

[44] Mantel N . The detection of disease clustering and a generalized regression approach. *Cancer Res* 1967;27:209–20. 6018555

[45] Reshef DN, Reshef YA, Finucane HK. et al. Detecting novel associations in large data sets. *Science* 2011;334:1518–24. 10.1126/science.1205438 22174245 PMC3325791

[46] Dixon P . VEGAN, a package of R functions for community ecology. *J Veg Sci* 2003;14:927–30. 10.1111/j.1654-1103.2003.tb02228.x

[47] Ji X, Tang J, Zhang J. Effects of salt stress on the morphology, growth and physiological parameters of Juglans microcarpa L. *Seedlings PLANTS-BASEL* 2022;11:2381. 10.3390/plants11182381 36145780 PMC9506368

[48] Chen C, Wu Y, Li J. et al. TBtools-II: a ‘one for all, all for one’bioinformatics platform for biological big-data mining. *Mol Plant* 2023;16:1733–42. 10.1016/j.molp.2023.09.010 37740491

[49] Deng Y, Jiang Y-H, Yang Y. et al. Molecular ecological network analyses. *BMC Bioinformatics* 2012;13:113. 10.1186/1471-2105-13-113 22646978 PMC3428680

[50] Parks DH, Imelfort M, Skennerton CT. et al. CheckM: assessing the quality of microbial genomes recovered from isolates, single cells, and metagenomes. *Genome Res* 2015;25:1043–55. 10.1101/gr.186072.114 25977477 PMC4484387

[51] Kanehisa M, Sato Y, Morishima K. BlastKOALA and GhostKOALA: KEGG tools for functional characterization of genome and metagenome sequences. *J Mol Biol* 2016;428:726–31. 10.1016/j.jmb.2015.11.006 26585406

[52] Chaumeil P-A, Mussig AJ, Hugenholtz P. et al. GTDB-Tk: a toolkit to classify genomes with the genome taxonomy database. *Bioinformatics* 2020;36:1925–7. 10.1093/bioinformatics/btz848 PMC7703759 31730192

[53] Seemann T . Prokka: rapid prokaryotic genome annotation. *Bioinformatics* 2014;30:2068–9. 10.1093/bioinformatics/btu153 24642063

[54] Kanehisa M, Sato Y, Kawashima M. KEGG mapping tools for uncovering hidden features in biological data. *Protein Sci* 2022;31:47–53. 10.1002/pro.4172 34423492 PMC8740838

[55] Aramaki T, Blanc-Mathieu R, Endo H. et al. KofamKOALA: KEGG Ortholog assignment based on profile HMM and adaptive score threshold. *Bioinformatics* 2020;36:2251–2. 10.1093/bioinformatics/btz859 31742321 PMC7141845

[56] Valdemarsen T, Kristensen E. Degradation of dissolved organic monomers and short-chain fatty acids in sandy marine sediment by fermentation and sulfate reduction. *Geochim Cosmochim Acta* 2010;74:1593–605. 10.1016/j.gca.2009.12.009

[57] Coskun OK, Gomez-Saez G, Beren M. et al. Quantifying genome-specific carbon fixation in a 750-meter deep subsurface hydrothermal microbial community. *FEMS Microbiol Ecol* 2024;100:fiae062. 10.1093/femsec/fiae062 38632042 PMC11075769

[58] Pan J, Xu W, Zhou Z. et al. Genome-resolved evidence for functionally redundant communities and novel nitrogen fixers in the deyin-1 hydrothermal field. *Mid-Atlantic Ridge Microbiome* 2022;10:8. 10.1186/s40168-021-01202-x 35045876 PMC8767757

[59] Garber AI, Ramirez GA, D’Hondt S. Genomic stasis over millions of years in subseafloor sediment. *Environ Microbiol* 2024;26:e16674. 10.1111/1462-2920.16674 39146976

[60] Jorgensen BB, Boetius A. Feast and famine - microbial life in the deep-sea bed. *Nat Rev Microbiol* 2007;5:770–81. 10.1038/nrmicro1745 17828281

[61] Marsland R, Cui W, Goldford J. et al. Available energy fluxes drive a transition in the diversity, stability, and functional structure of microbial communities. *PLoS Comput Biol* 2019;15:e1006793. 10.1371/journal.pcbi.1006793 30721227 PMC6386421

[62] Karimi B, Maron PA, Boure NC-P. et al. Microbial diversity and ecological networks as indicators of environmental quality. *Environ Chem Lett* 2017;15:265–81. 10.1007/s10311-017-0614-6

[63] Liu B, Zheng Y, Wang X. et al. Active dark carbon fixation evidenced by 14C isotope assimilation and metagenomic data across the estuarine-coastal continuum. *Sci Total Environ* 2024;914:169833. 10.1016/j.scitotenv.2023.169833 38190922

[64] Mootapally C, Sharma P, Dash S. et al. Microbial drivers of biogeochemical cycles in deep sediments of the Kathiawar peninsula gulfs of India. *Sci Total Environ* 2025;965:178609. 10.1016/j.scitotenv.2025.178609 39892243

[65] Chi X, Zhao Z, Han Q. et al. Insights into autotrophic carbon fixation strategies through metagonomics in the sediments of seagrass beds. *Mar Environ Res* 2023;188:106002. 10.1016/j.marenvres.2023.106002 37119661

[66] Chen X, Liu J, Zhu X-Y. et al. Phylogenetically and metabolically diverse autotrophs in the world’s deepest blue hole. *ISME Commun* 2023;3:117. 10.1038/s43705-023-00327-4 37964026 PMC10645885

[67] Jiang Q, Jing H, Jiang Q. et al. Insights into carbon-fixation pathways through metagonomics in the sediments of deep-sea cold seeps. *Mar Pollut Bull* 2022;176:113458. 10.1016/j.marpolbul.2022.113458 35217425

[68] Ruiz-Fernandez P, Ramirez-Flandes S, Rodriguez-Leon E. et al. Autotrophic carbon fixation pathways along the redox gradient in oxygen-depleted oceanic waters. *Environ Microbiol Rep* 2020;12:334–41. 10.1111/1758-2229.12837 32202395 PMC7318340

[69] Berg IA, Kockelkorn D, Ramos-Vera WH. et al. Autotrophic carbon fixation in archaea. *Nat Rev Microbiol* 2010;8:447–60. 10.1038/nrmicro2365 20453874

[70] Seitzinger S, Harrison JA, Bohlke JK. et al. Denitrification across landscapes and waterscapes: a synthesis. *Ecol Appl* 2006;16:2064–90. 10.1890/1051-0761(2006)016[2064:DALAWA]2.0.CO;2 17205890

[71] Marzocchi U, Trojan D, Larsen S. et al. Electric coupling between distant nitrate reduction and sulfide oxidation in marine sediment. *ISME J* 2014;8:1682–90. 10.1038/ismej.2014.19 24577351 PMC4817607

[72] Adam PS, Borrel G, Gribaldo S. Evolutionary history of carbon monoxide dehydrogenase/acetyl-CoA synthase, one of the oldest enzymatic complexes (vol 115, Pg E1166, 2018). *Proc Natl Acad Sci USA* 2018;115:E5836–7. 10.1073/pnas.1807540115 29358391 PMC5819426

[73] Ruiz-Blas F, Bartholomaeus A, Yang S. et al. Metabolic features that select for Bathyarchaeia in modern ferruginous lacustrine subsurface sediments. *Isme Commun* 2024;4:ycae112. 10.1093/ismeco/ycae112 39660009 PMC11631310

[74] Yue X-L, Xu L, Cui L. et al. Metagenome-based analysis of carbon-fixing microorganisms and their carbon-fixing pathways in deep-sea sediments of the southwestern Indian Ocean. *Mar Genomics* 2023;70:101045. 10.1016/j.margen.2023.101045 37245381

[75] Zampieri G, Santinello D, Palu M. et al. Core cooperative metabolism in low-complexity CO2-fixing anaerobic microbiota. *ISME J* 2025;19:wraf017. 10.1093/ismejo/wraf017 39893570 PMC11844248

[76] Taubert M, Overholt WA, Heinze BM. et al. Bolstering fitness via CO2 fixation and organic carbon uptake: mixotrophs in modern groundwater. *ISME J* 2022;16:1153–62. 10.1038/s41396-021-01163-x 34876683 PMC8941145

[77] Ragsdale SW . Enzymology of the wood-Ljungdahl pathway of acetogenesis. *Ann N Y Acad Sci* 2008;1125:129–36. 10.1196/annals.1419.015 18378591 PMC3040112

[78] Huang H, Wang S, Moll J. et al. Electron bifurcation involved in the energy metabolism of the Acetogenic bacterium Moorella thermoacetica growing on glucose or H2 plus CO2. *J Bacteriol* 2012;194:3689–99. 10.1128/JB.00385-12 22582275 PMC3393501

[79] Schuchmann K, Mueller V. Autotrophy at the thermodynamic limit of life: a model for energy conservation in acetogenic bacteria. *Nat Rev Microbiol* 2014;12:809–21. 10.1038/nrmicro3365 25383604

[80] Hess V, Poehlein A, Weghoff MC. et al. A genome-guided analysis of energy conservation in the thermophilic, cytochrome-free acetogenic bacterium Thermoanaerobacter kivui. *BMC Genomics* 2014;15:1139. 10.1186/1471-2164-15-1139 25523312 PMC4320612

[81] Lever MA . Acetogenesis in the energy-starved deep biosphere - a paradox? *Front Microbiol* 2012;3:284. 10.3389/fmicb.2011.00284 22347874 PMC3276360

[82] Orsi WD, Schink B, Buckel W. et al. Physiological limits to life in anoxic subseafloor sediment. *FEMS Microbiol Rev* 2020;44:219–31. 10.1093/femsre/fuaa004 32065239 PMC7269680

[83] Lau MCY, Kieft TL, Kuloyo O. et al. An oligotrophic deep-subsurface community dependent on syntrophy is dominated by sulfur-driven autotrophic denitrifiers. *Proc Natl Acad Sci USA* 2016;113:E7927–36. 10.1073/pnas.1612244113 27872277 PMC5150411

[84] Kerou M, Ponce-Toledo RI, Zhao R. et al. Genomes of Thaumarchaeota from deep sea sediments reveal specific adaptations of three independently evolved lineages. *ISME J* 2021;15:2792–808. 10.1038/s41396-021-00962-6 33795828 PMC8397731

[85] Peres FV, Paula FS, Bendia AG. et al. Assessment of prokaryotic communities in Southwestern Atlantic deep-sea sediments reveals prevalent methanol-oxidising Methylomirabilales. *Sci Rep* 2023;13:12782. 10.1038/s41598-023-39415-9 37550336 PMC10406867

[86] Nancharaiah YV, Lens PNL. Ecology and biotechnology of selenium-respiring bacteria. *Microbiol Mol Biol Rev* 2015;79:61–80. 10.1128/MMBR.00037-14 25631289 PMC4402961

[87] Wasmund K, Schreiber L, Lloyd KG. et al. Genome sequencing of a single cell of the widely distributed marine subsurface Dehalococcoidia, phylum Chloroflexi. *ISME J* 2014;8:383–97. 10.1038/ismej.2013.143 23966099 PMC3906807

[88] Kube M, Beck A, Zinder SH. et al. Genome sequence of the chlorinated compound respiring bacterium Dehalococcoides species strain CBDB1. *Nat Biotechnol* 2005;23:1269–73. 10.1038/nbt1131 16116419

[89] Seshadri R, Adrian L, Fouts DE. et al. Genome sequence of the PCE-dechlorinating bacterium Dehalococcoides ethenogenes. *Science* 2005;307:105–8. 10.1126/science.1102226 15637277

[90] Wasmund K, Cooper M, Schreiber L. et al. Single-cell genome and group-specific dsrAB sequencing implicate marine members of the class Dehalococcoidia (phylum Chloroflexi) in Sulfur cycling. *MBio* 2016;7:e00266–16. 10.1128/mBio.00266-16 27143384 PMC4959651

[91] Huang J-M, Baker BJ, Li J-T. et al. New microbial lineages capable of carbon fixation and nutrient cycling in Deep-Sea sediments of the northern South China Sea. *Appl Environ Microbiol* 2019;85:e00523–19. 10.1128/AEM.00523-19 31126943 PMC6643227

[92] Wang Y, Gao Z-M, Li J-T. et al. Draft genome of an Aerophobetes bacterium reveals a facultative lifestyle in deep-sea anaerobic sediments. *Sci Bull* 2016;61:1176–86. 10.1007/s11434-016-1135-6

[93] Dong X, Greening C, Rattray JE. et al. Metabolic potential of uncultured bacteria and archaea associated with petroleum seepage in deep-sea sediments. *Nat Commun* 2019;10:1816. 10.1038/s41467-019-09747-0 31000700 PMC6472368

