# Supplementary material for: Vertically stratified carbon fixation and coupling processes in deep-sea sediment
Source: ISME Commun. 2025 Dec 18;5(1):ycaf242. doi: 10.1093/ismeco/ycaf242 (PMC12766754; doi:10.1093/ismeco/ycaf242)
Supplement: supplementary_figures_ycaf242 [file supplementary_figures_ycaf242.docx]

**Vertically stratified carbon fixation and coupling processes in deep-sea sediment**

**Hai Shi**^1^**, Xiaotong Zhang**^1^**, Liyan Liu**^1^**, Fabiano Thompson**^2^**, Xueqi Li^1^, Haowei Sun^1^, Huichao Mi^1^, Xiao-Hua Zhang**^1,3,4^ **and Yunhui Zhang**^4*^

^1^ Frontiers Science Center for Deep Ocean Multispheres and Earth System, and College of Marine Life Sciences, Ocean University of China, Qingdao, China

^2^ Institute of Biology and Coppe, Federal University of Rio de Janeiro (UFRJ), Rio, 21941-599, Brazil

^3^ Laboratory for Marine Ecology and Environmental Science, Qingdao Marine Science and Technology Center, Qingdao, China

^4^ Key Laboratory of Evolution & Marine Biodiversity (Ministry of Education) and Institute of Evolution & Marine Biodiversity, Ocean University of China, Qingdao, China

Corresponding author: Yunhui Zhang, [zhangyunhui@ouc.edu.cn](mailto:zhangyunhui@ouc.edu.cn)

**Supplementary figures**





**Figure S1:** Sampling site in the SCS sediments.


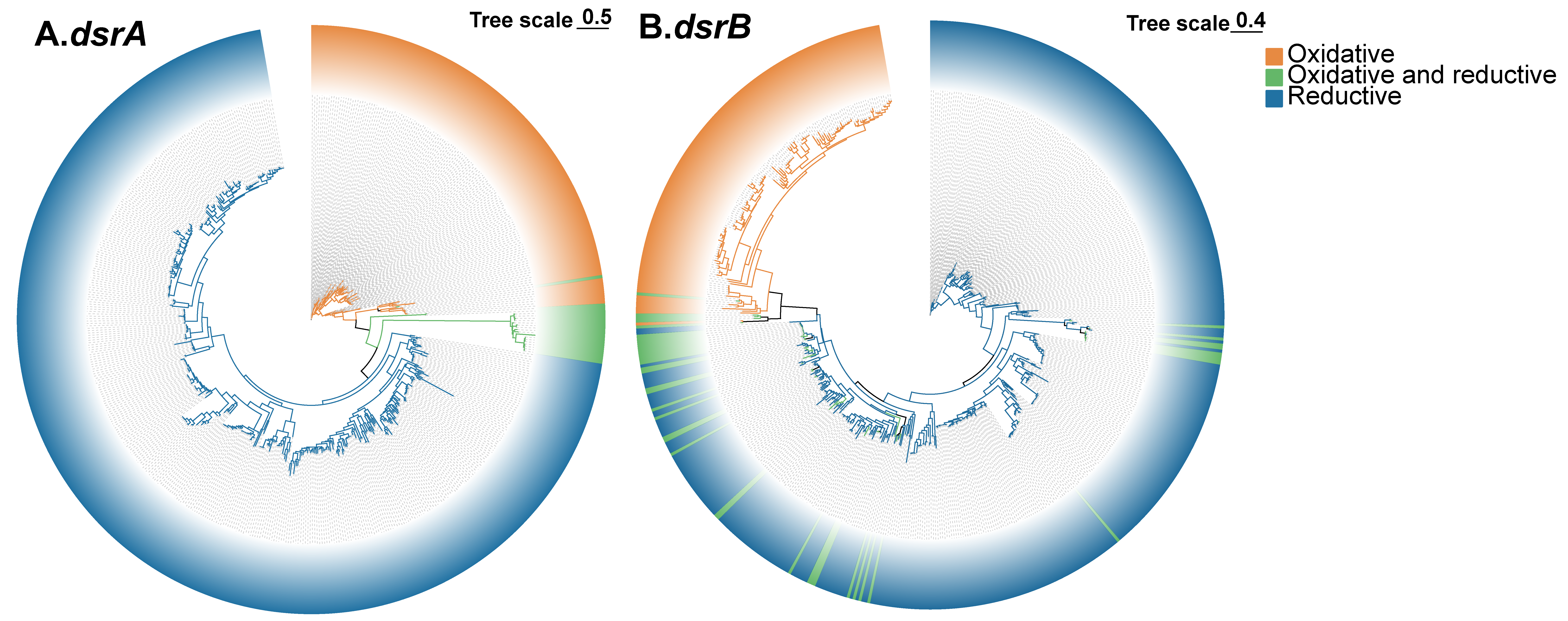


**Figure S2:** Maximum likelihood trees constructed based on the *dsrA* (A) and *dsrB* (B) retrieved from SCS sediment metagenomes. Blue indicates sequences matched to *dsrAB*, orange indicates sequences matched to *rdsrAB*, and green represents sequences matched to both *dsrAB* and *rdsrAB.*

**
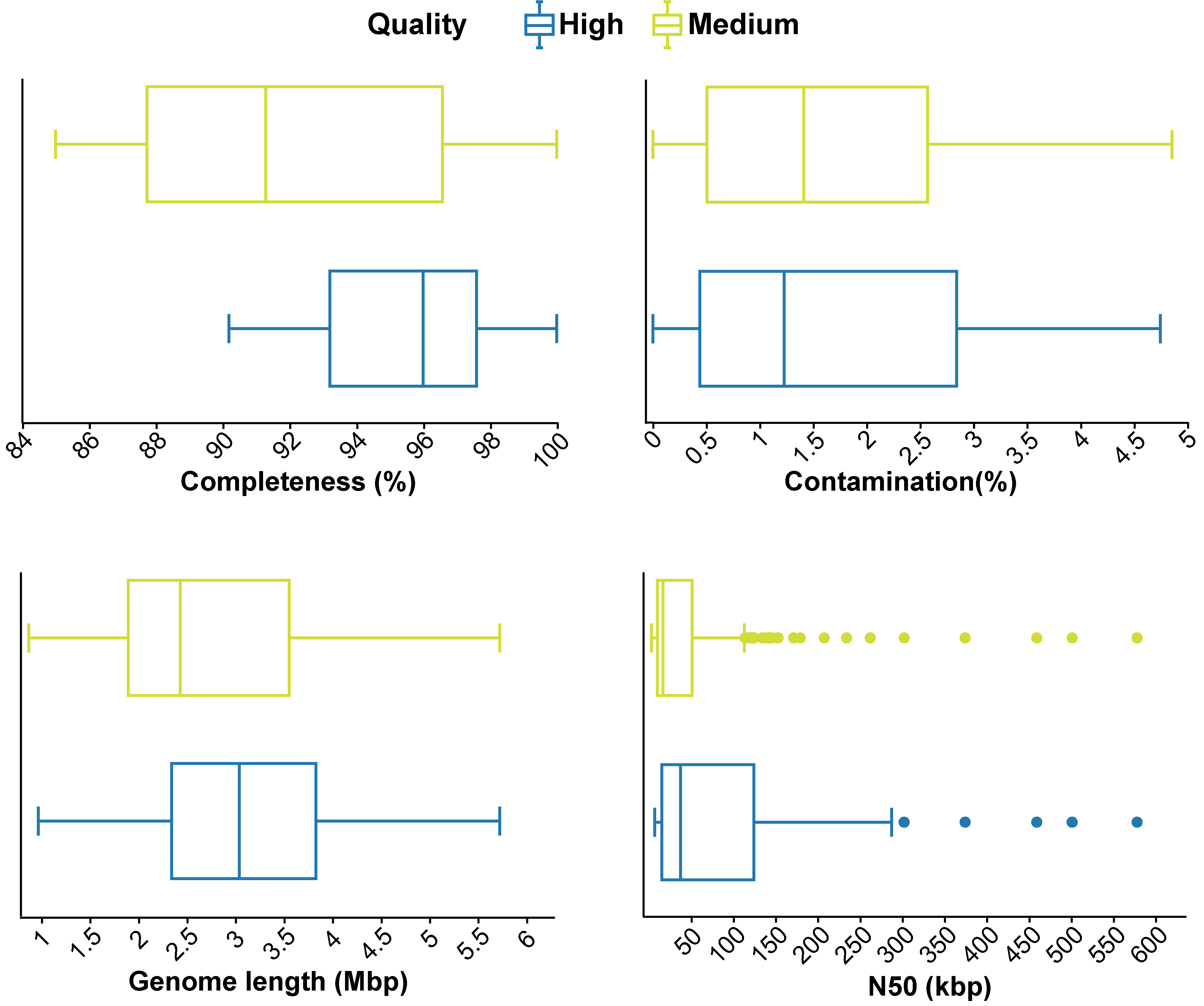
**

**Figure S3:** General characteristics of all retrieved MAGs including N50 (kbp), genome length (Mbp), completeness (%) and contamination (%)). The MAGs are grouped into medium quality (N = 336 MAGs) and high quality (N = 205 MAGs), respectively.

**
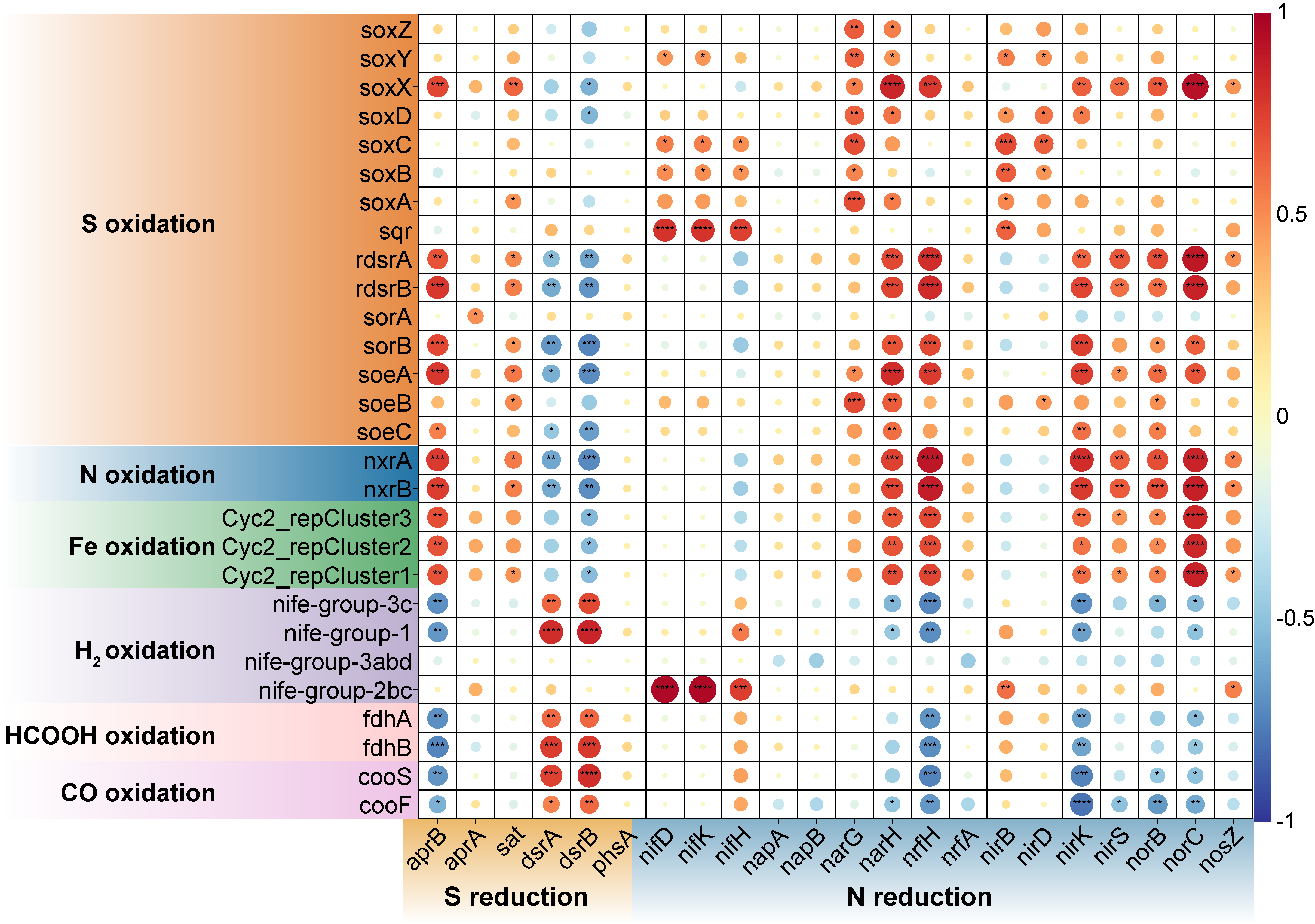
**

**Figure S4:** The correlation between genes involved in main oxidation and reduction processes supporting carbon fixation.

**
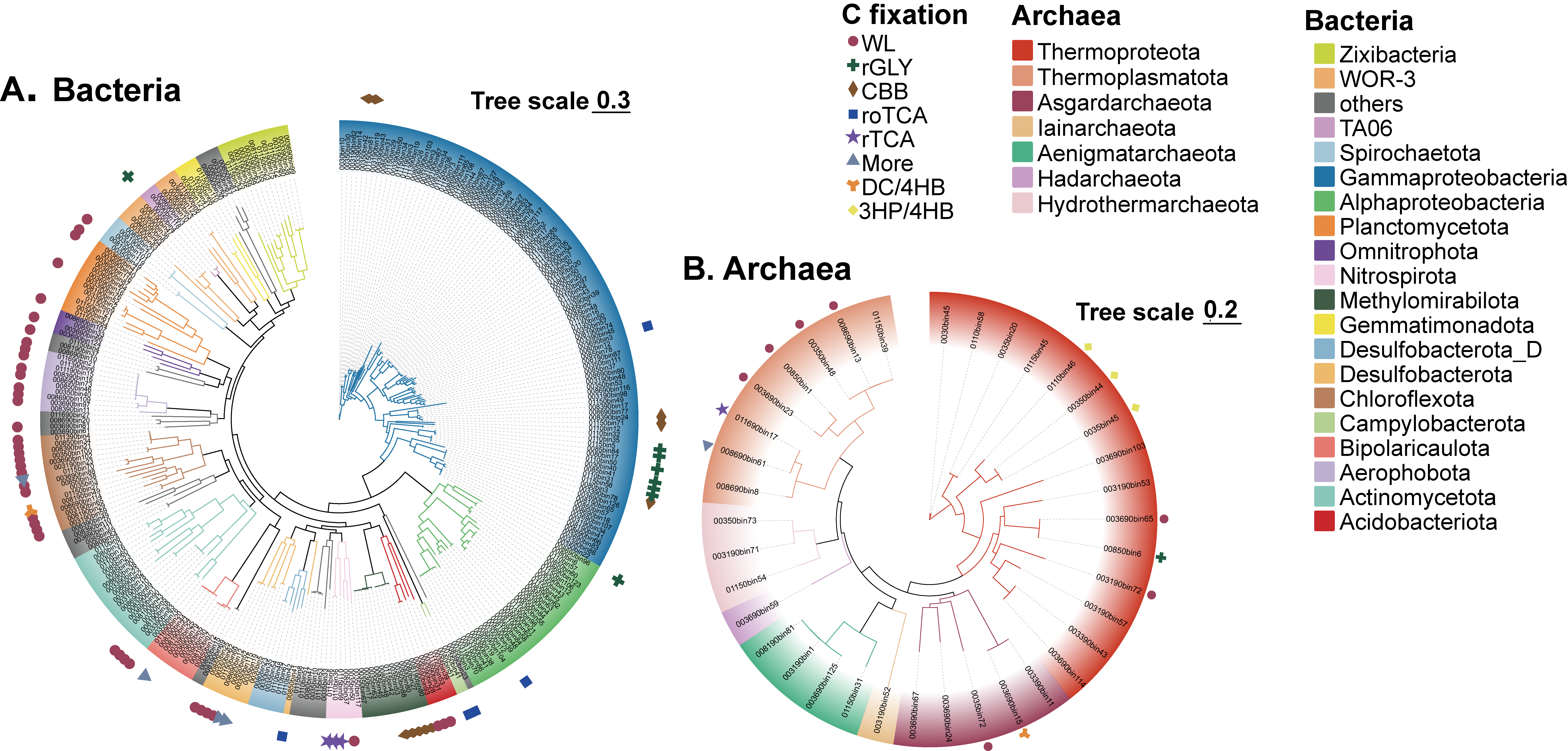
**

**Figure S5:** Maximum likelihood phylogenetic tree of the bacterial (A) and archaea (B) MAGs. The colors of the branches and leaves correspond to the phylum-level taxonomic affiliations of the MAGs. Different shapes and colored blocks indicate the carbon fixation types associated with the MAGs.

**
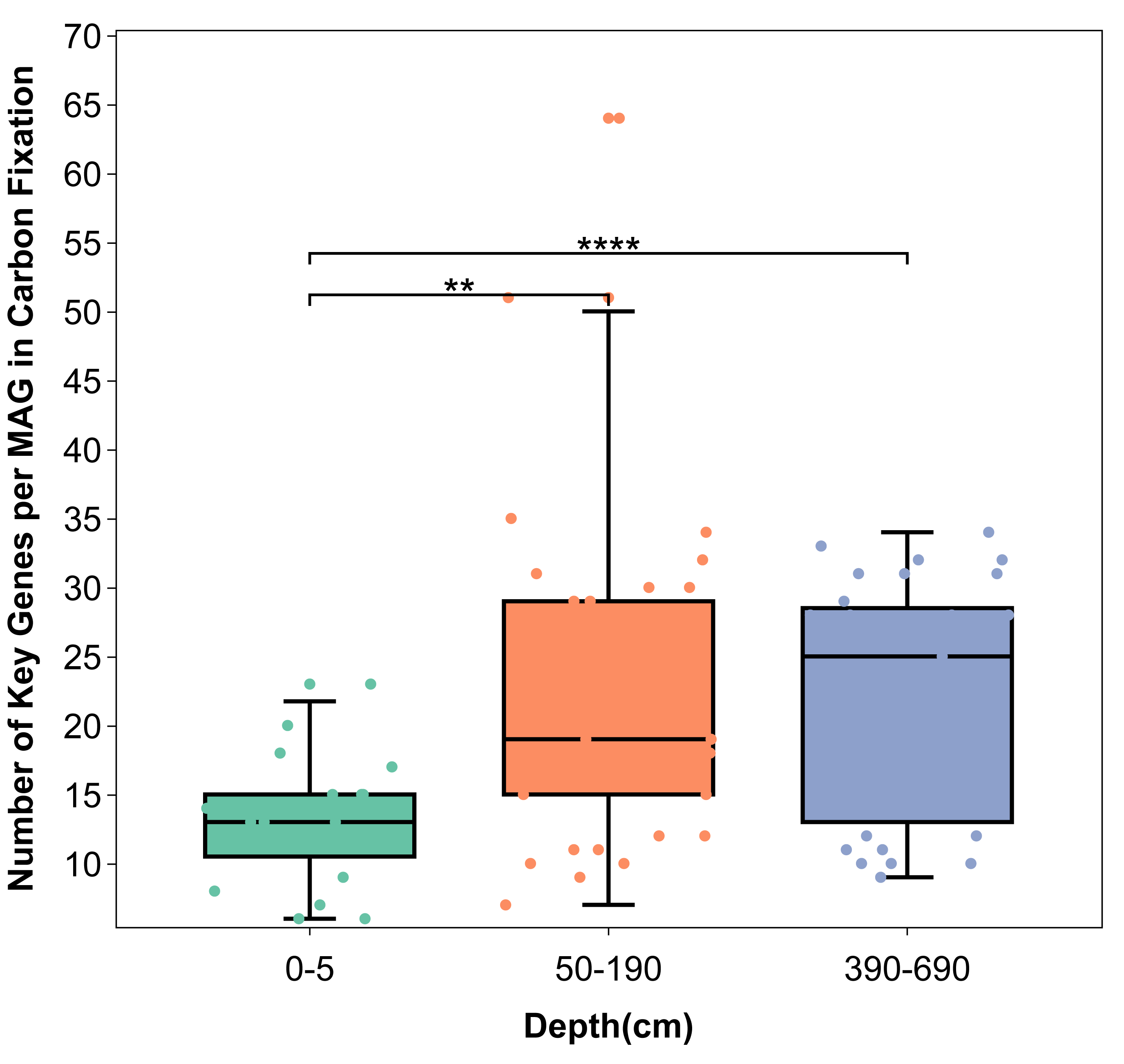
**

**Figure S6:** Number of key carbon-fixation genes detected in MAGs across sediment depths (t-test). Significance: * P < 0.05; ** P < 0.01; *** P < 0.001; **** P < 0.0001.

**
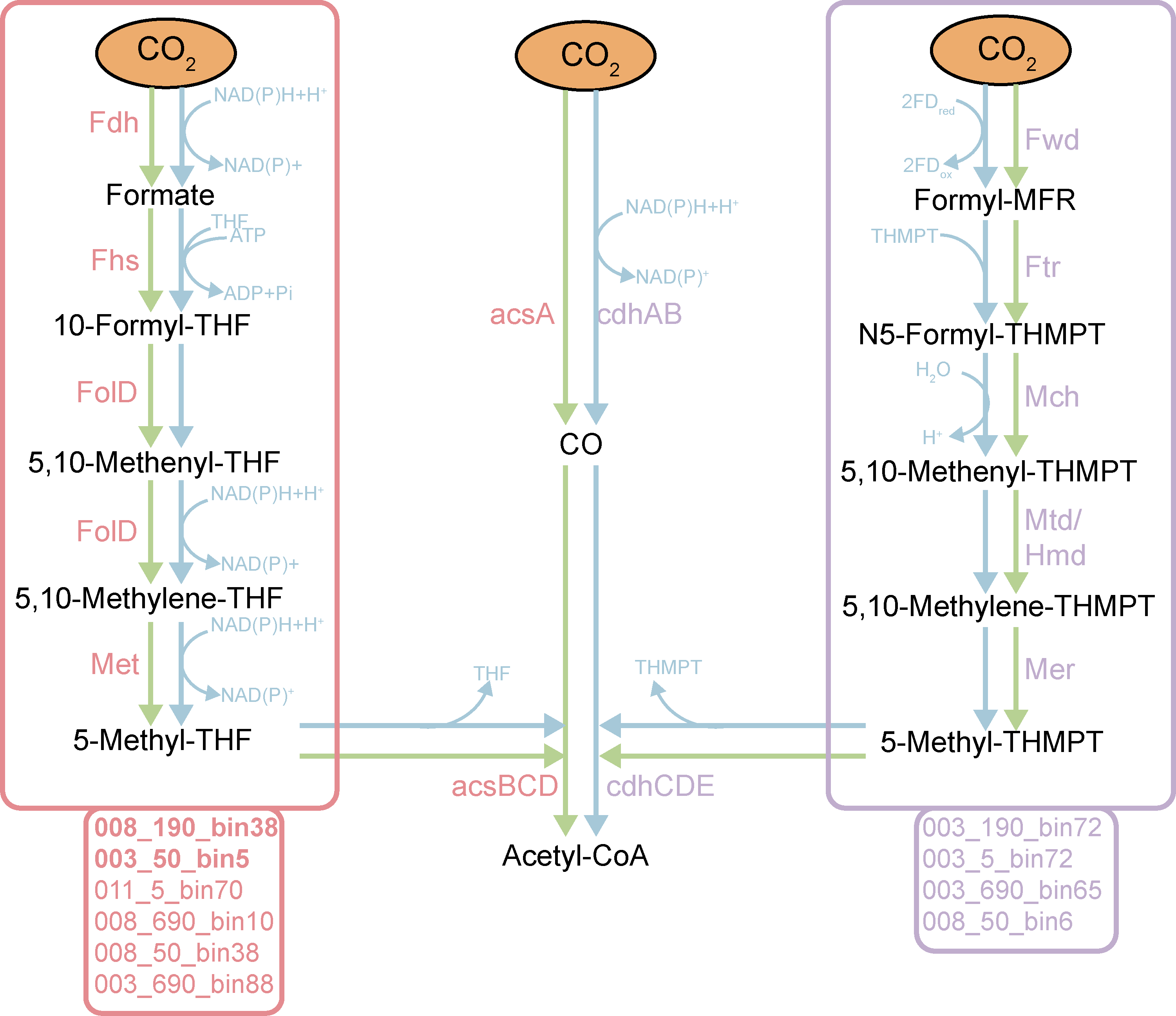
**

**Figure S7:** Summary of WL pathway in carbon-fixing MAGs. A MAG is considered to possess a complete WL pathway only if it contains all genes required to encode a full enzymatic route for the metabolic process. All MAGs shown here harbor genes related to the carbonyl branch. Red and purple colors indicate H₄MPT- and THF-dependent methyl branches, respectively. Bold red MAGs possess a complete H₄MPT-type methyl branch, while the regular red ones lack the fdh gene at the initial step but encode alternative formate dehydrogenase subunits, suggesting potential functional replacement. In total, ten MAGs were predicted to contain a complete WL pathway. Purple-colored MAGs combine the THF-type methyl branch with the carbonyl branch to form a complete WL carbon fixation route.

**
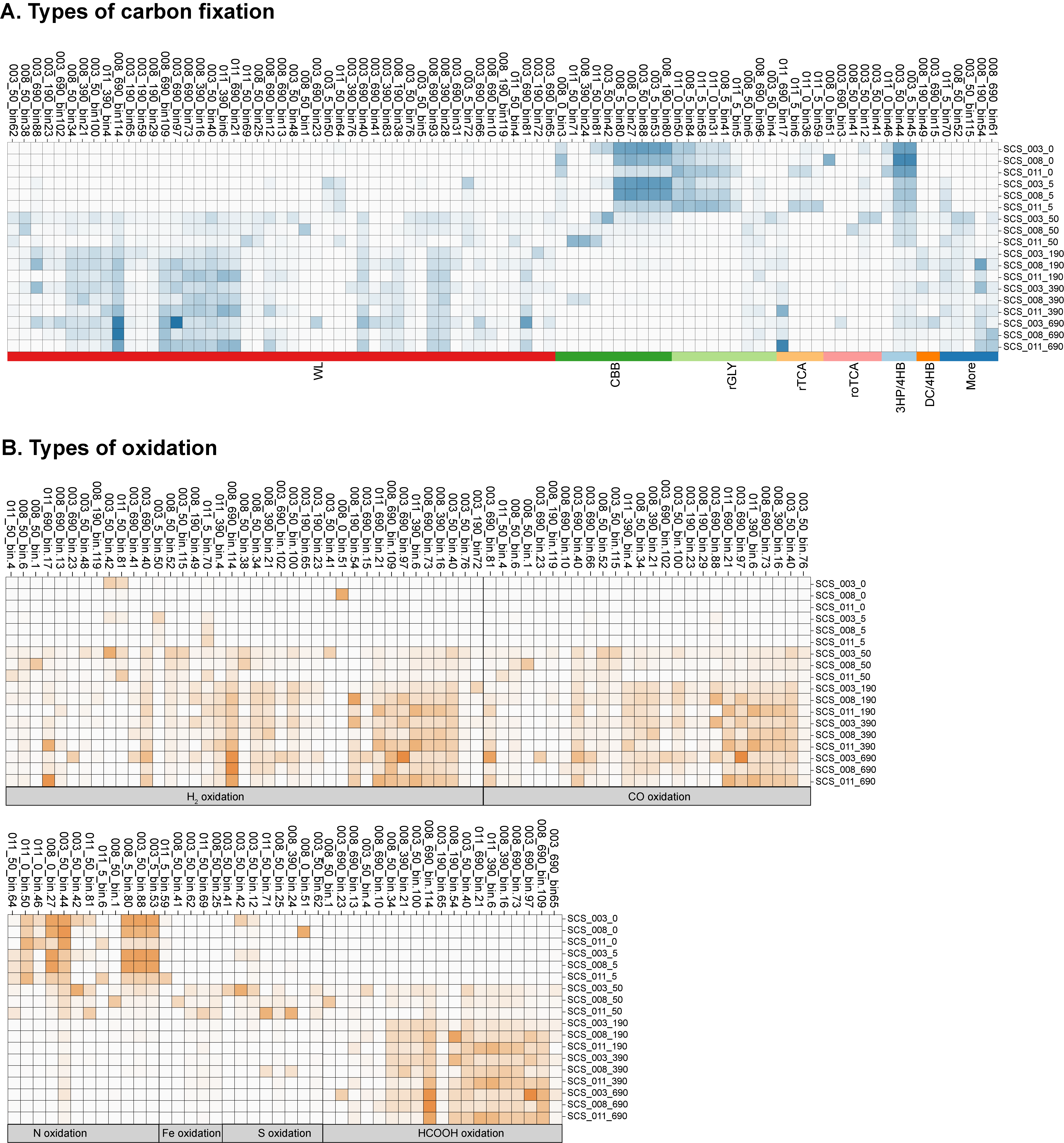
**

**Figure S8:** The relative abundance (RPKM) of MAGs with different carbon fixation pathway (A) and oxidation types (B) across all samples.

**
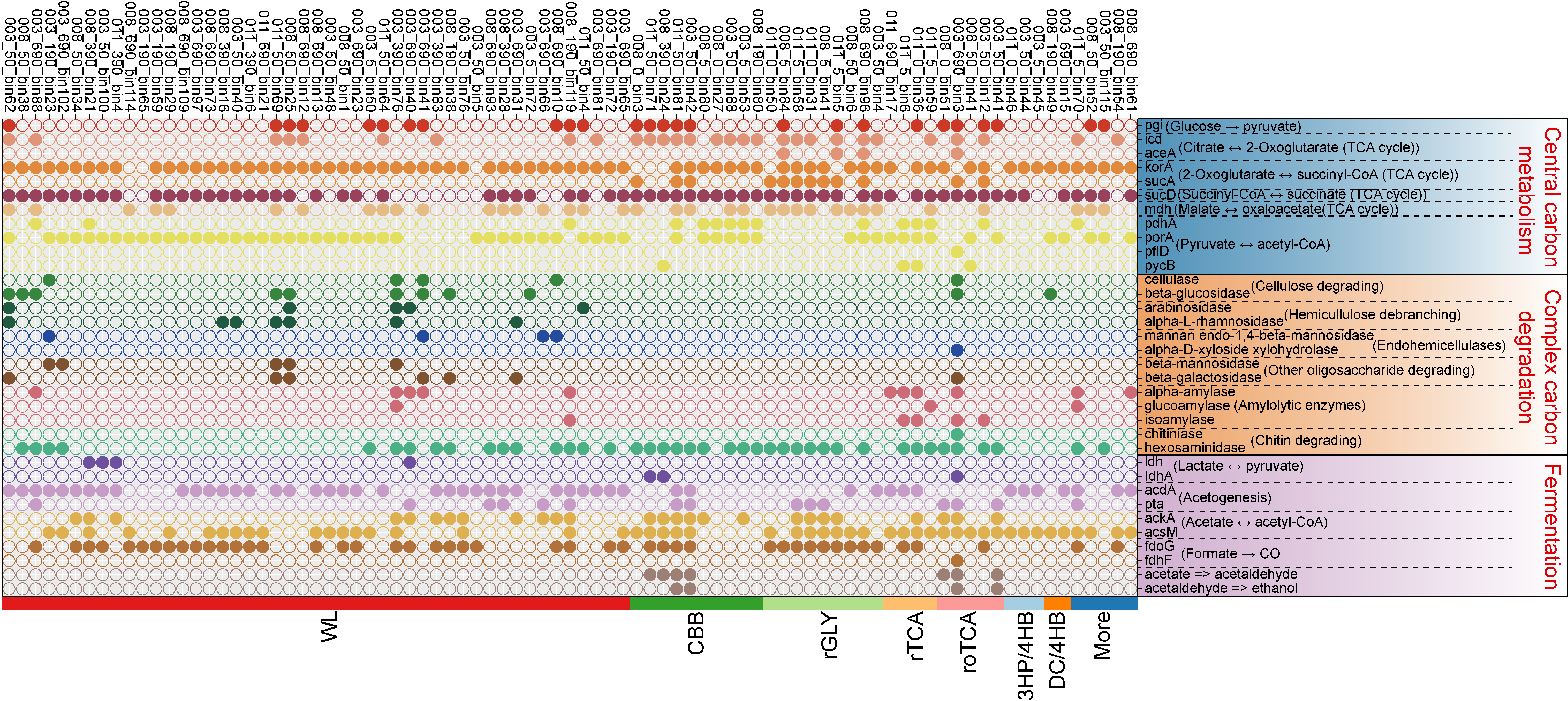
Figure S9:** Organic substrate utilization potential of 85 carbon-fixing MAGs. Circle colors indicate the presence or absence of corresponding functional genes related to organic matter metabolism. MAGs are divided into two groups based on whether they possess conventional inorganic oxidation pathways. Colored bars at the bottom denote different types of carbon fixation pathways.


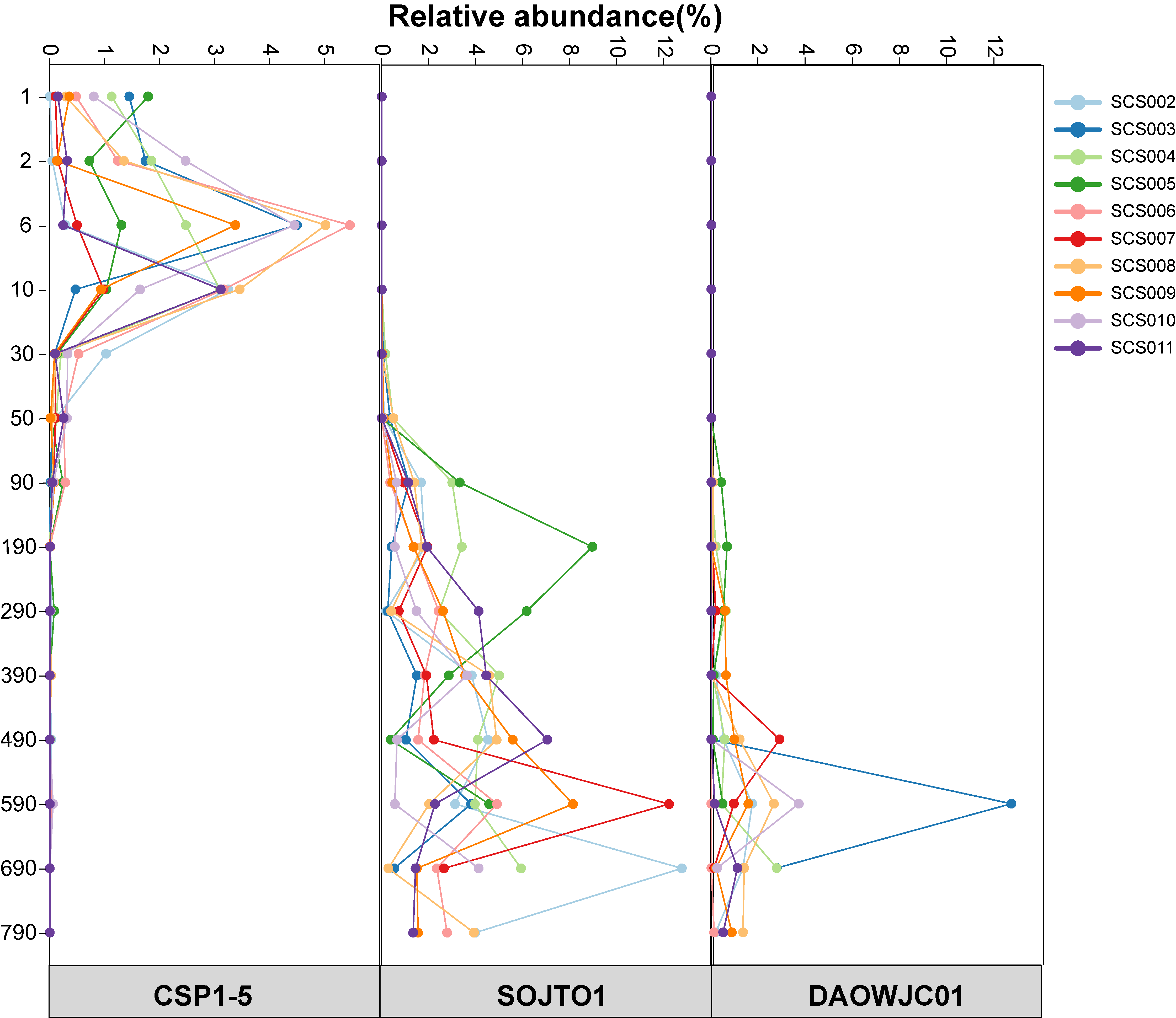


**Figure S10:** Relative abundance analysis based on 16S amplicon sequencing. The x-axis represents the relative abundance of 16S rRNA genes, and the y-axis indicates sediment depth. Lines in different colors correspond to different sampling sites based on previous studies.
